# Modelling Plasmid-Mediated Horizontal Gene Transfer in Biofilms

**DOI:** 10.1007/s11538-024-01289-x

**Published:** 2024-04-25

**Authors:** Julien Vincent, Alberto Tenore, Maria Rosaria Mattei, Luigi Frunzo

**Affiliations:** 1 Department of Mathematics and Applications “Renato Caccioppoli”, University of Naples Federico II, Via Cintia 26, 80126 Monte S. Angelo, Naples, Italy; 2 Microbial Ecology Laboratory, University of Galway, University Road, Galway, H91 TK33 Ireland

**Keywords:** Horizontal gene transfer, Biofilms, Plasmid conjugation, Antimicrobial resistence, Free boundary problem, Nonlocal PDEs

## Abstract

In this study, we present a mathematical model for plasmid spread in a growing biofilm, formulated as a nonlocal system of partial differential equations in a 1-D free boundary domain. Plasmids are mobile genetic elements able to transfer to different phylotypes, posing a global health problem when they carry antibiotic resistance factors. We model gene transfer regulation influenced by nearby potential receptors to account for recipient-sensing. We also introduce a promotion function to account for trace metal effects on conjugation, based on literature data. The model qualitatively matches experimental results, showing that contaminants like toxic metals and antibiotics promote plasmid persistence by favoring plasmid carriers and stimulating conjugation. Even at higher contaminant concentrations inhibiting conjugation, plasmid spread persists by strongly inhibiting plasmid-free cells. The model also replicates higher plasmid density in biofilm’s most active regions.

## Introduction

The spread of antibiotic resistance is becoming an increasingly important global healthcare problem, as it affects our ability to treat bacterial infections. Selective pressure of antibiotics could induce the spread of antibiotic resistant bacteria and antibiotic resistance genes (ARG) (Wright 2010). ARG and metal resistance genes (MRG) spread and control is a major public health issue and an emerging challenge to address (Pruden 2014).There is also a growing concern that metals participate in the selective pressure responsible for the proliferation of ARG (Wang et al. 2017), making metal contamination a widespread and recalcitrant selective pressure driving the maintenance and spread of antibiotic resistance (Baker-Austin et al. 2006; Shi et al. 2022). These co-selection mechanisms to metals and antibiotics have been widely documented in the literature (Pal et al. 2015; Chen et al. 2019; Zhang et al. 2018) and can be co-resistance, i.e. the presence of different resistance determinants on the same gene, or cross-resistance, i.e. the resistance to antibiotics and metals comes from the same genetic determinant (Baker-Austin et al. 2006). These co-selection mechanisms participate in the widespread of antibiotic and metal resistance, which dissemination is largely associated to extrachromosomal genetic elements called plasmids. Plasmids are able to transfer to new host cells through horizontal gene transfer even when they are phylogenetically distant. More specifically, conjugation plays a crucial role in the ecological success of plasmids in bacterial communities, as approximately half of plasmids are conjugation-able (Smillie et al. 2010). In the case of co-resistance or cross-resistance, the plasmid carrying ARG also carries metal resistance genes (MRG) and successful transfer of resistance genes will influence the resistance of the recipient to metals and antibiotics. In Baker-Austin et al. (2006), various instances of shared resistance mechanisms are provided. For example, modifying cellular components to alter their sensitivity to toxic elements not only enhances cell resistance to Hg, Zn, and Cu but also affects the effectiveness of antibiotics like ciprofloxacin, $\upbeta$-lactams, trimethoprim, and rifampicin (Barkay et al. 2003; Roberts 2005).

Conjugation depends on the physical contact between cells, which makes biofilms a preferential environment (Abe et al. 2020; Savage et al. 2013). Biofilms are polymicrobial aggregates encased in a matrix of self-produced extracellular polymeric substances (EPS) (Flemming et al. 2016) offering a protective habitat to bacteria from environmental hazards, such as nutrient depletion, or the presence of toxic stressors (Flemming and Wingender 2010). There is experimental evidence of conjugative transfer being extensively present in wastewater treatment plants (Zhang and Li 2011; Guo et al. 2017), thus contributing to the global spreading of microbial resistance in the environment.

Experimental evidence suggests that the process of plasmid conjugation within biofilms is limited to subcolonies within the biofilm (Stalder and Top 2016). Instead of a wave-like phenomena that would sweep the entire biofilm and allow successful gene transfer, horizontal gene transfer (HGT) allows the presence of subpopulations of donor. This is consistent with the high heterogeneity of biofilms, where different niches exposed to different selective pressures lead to highly heterogeneous populations, conferring an evolutionary advantage (Ciofu et al. 2022). The nature of the signals regulating conjugation is yet to be elucidated for complex matrices such as biofilms, but experimental studies have demonstrated the implication of regulators such as quorum sensing, nutrients gradient, the SOS response or extracytoplasmic stress (Beaber et al. 2004; Frost and Koraimann 2010). Moreover, the very low or null growth rate of the cells located deep within the biofilm could be associated with a limited HGT (Stalder and Top 2016). Resistance to antibiotics or metals by carrying a plasmid is associated with a fitness cost for the bacteria (Ciofu et al. 2022); carrying a resistance plasmid or having conjugative transfer genes expressed is not always beneficial, thus underlining the importance of selective pressure. Expressing or not the conjugative transfer gene alleviates, or even eliminates the fitness cost: not all donors are transfer competent cells, phenomenon governed by the expression of the *tra* operon (Koraimann and Wagner 2014). A consequent fitness cost for the host bacteria is due to the expression of the *tra* genes on the conjugative plasmid, thus explaining the importance of the regulation of this expression (Baltrus 2013) for the fitness of the population. Therefore, strategies come with the expression of *tra* genes, in order to minimize fitness cost for the population. The default setting for gene expression is OFF and under specific circumstances, only a fraction of the population carrying a conjugative plasmid will transit to the ON stage. It is still unclear if this process is stochastic or depends on the physiological state of the biofilm or individual cells, i.e. metabolic conditions, cell age, cellular fitness or the position of individual cells within the biofilm (Koraimann and Wagner 2014). This strategy allows the persistence of resistance genes even in a situation without continuous selective pressure as plasmid-bearing bacteria can continue to grow with reduced or even without any restriction coming from a fitness cost.

Given the complexity of these processes, mathematical modelling is a widely used approach to simulate the behavior of complex bacterial communities. Just as laboratory systems do not represent the actual conditions in nature, simulation models simplify systems under sets of assumptions in order to investigate desired aspects of its behavior. HGT and conjugation have been extensively studied from a mathematical modelling perspective in the literature; Leclerc et al. (2019) proposes a systematic review of modelling studies focusing on HGT of antimicrobial resistance in bacterial populations. Although this review does not focus on biofilm models, it summarizes how conjugation is modelled in the literature: in most cases conjugation is modelled as a mass action process (Leclerc et al. 2019). In the studies presented in this review, both deterministic (Cazer et al. 2017; Knopoff and Sánchez Sansó 2016; Kneis et al. 2019; Xu et al. 2018; Zwanzig et al. 2019; Gothwal and Thatikonda 2018) and stochastic (Volkova et al. 2013; Zhong et al. 2012; Freese et al. 2014; Campos et al. 2019) approaches have been considered. The deterministic studies tracked the evolution of different subpopulations (sensitive, resistant, etc.) with ordinary differential equations, without integrating the influence of space in the model. In Szomolay et al. (2005), the authors have modelled the diffusion-limited protection mechanism of cells within a biofilm, without considering conjugation but adaptation of cells when exposed to an antibiotic. In Arya et al. (2021), a deterministic and a stochastic approach are compared to model resistance and predict the minimal metal concentration co-selecting for antibiotic resistance plasmids. They model conjugation as a Sensitive-Infected-Resistant/Recovered (SIR) model for plasmid transfer.

The objective of this study is to enhance the current modelling approaches for the spread and persistence of plasmid-borne resistance in bacterial biofilms by considering the impact of environmental factors on conjugation. This encompasses factors such as nutrient availability, recipient-sensing mechanisms, and selective pressure, with particular emphasis on co-resistance and cross-resistance. To this aim, we present a novel function that models the regulation of gene expression governing plasmid conjugation within a growing biofilm. The model consists of a nonlocal system of partial integro-differential equations with a convolution integral regulating the transfer genes expression to account for recipient-sensing; i.e. its dependence on the presence of potential receptors around a donor. Vertical gene transfer (VGT) during cell replication is also included, allowing to model how environmental conditions can favor plasmid-carrying cells despite a lower growth rate due to the cost of plasmid maintenance. Abiotic factors that regulate gene expression are also included: plasmid-free density, bacterial fitness and selective pressure from metals and antibiotics. The numerical simulations investigate plasmid ecology under different conditions, including how subinhibitory concentrations of trace metals can favor the spread of antibiotic resistance in the case of co-resistance.

The paper is organised as follows: in Sect. 2, the model equations and underlying assumptions are laid out. Section 3 discusses the existence and uniqueness of the solution. Section 4 describes the numerical methods and the numerical investigations performed with their biological implications. Finally, conclusions and future perspectives are reported in Sect. 5.

## Mathematical Model

In this section, we present a mathematical model able to track the dynamics of a biofilm ecosystem while including the transfer of mobile genetic elements giving metal and/or antibiotic resistance. A particular attention is dedicated to the processes regulating the transfer of these genetic elements and how they affect the population fitness as a whole, and the biotic and abiotic factors regulating the gene transfer. The biofilm is modelled as a one dimensional free-boundary domain of thickness *L*(*t*), growing in the *z* direction, perpendicularly to the substratum $z=0$. We consider its growth depends on one limiting substrate *N*—i.e. Dissolved Organic Carbon (DOC)—and is affected by antibiotic *A* and metal *M*. Their concentrations at position *z* and time *t* are denoted $S_N$, $S_A$ and $S_M$. The biofilm is constituted of seven solid phase components:

- plasmid-free bacteria *F*: bacteria with basal growth rate and basal sensitivity to toxic stressors.
- plasmid-carrying bacteria *P*: bacteria with a resistance plasmid. Their growth rate is reduced by a fitness cost $\lambda _1$, due to the energy cost of plasmid maintenance. This metabolic disadvantage is balanced by a higher resistance to toxic stressors given by the plasmid.
- plasmid-carrying bacteria expressing the *tra* gene for conjugation *C*: bacteria able to transmit plasmid via conjugation. Their growth rate is reduced by a fitness cost $\lambda _2>\lambda _1$ due to plasmid maintenance and additional gene expression allowing conjugation. They have the same resistance to toxic stressors as *P*.
- extracellular polymeric substances (*EPS*) are mainly polysaccharides, proteins, nucleic acids and lipids. They participate in the structure integrity of the biofilm and are produced during metabolic growth.
- inert biomass *I* is produced by decay of active microbial species.
- transconjugants *T*: transitory state of bacteria having just received a plasmid through conjugation. They do not divide nor decay and go back to normal state *P* after a short time with a rate $\uptau _T$.
- exhausted donors *D*: transitory state of bacteria having just transmitted a plasmid through conjugation, as conjugation copies the plasmid into the receptor: the donor still carries copies after conjugation. They do not divide nor decay and go back to normal state *P* after a short time with a rate $\uptau _D$.

**Fig. 1:**
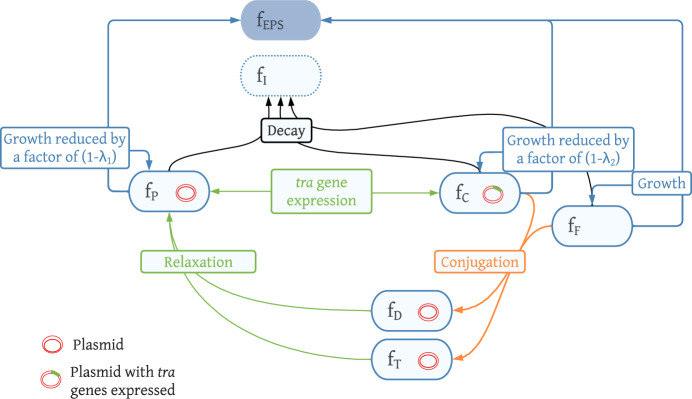
Schematic representation of the model biological pathways. Created in Lucidchart (www.lucidchart.com)

Solid-phase components are expressed in terms of volume fractions ${{\textbf {f}}}=(f_F,f_P,f_C,f_{EPS},f_I,f_T,f_D)$, with $f_i(z,t), i\in J_f=\{F,P,C,EPS,I,T,D\}$ or concentrations $X_i=f_i\rho$, where $\rho$ is the biofilm density, assumed to be constant and equal for all constituents *i*. The volume fractions are constrained to add up to unity $\sum _{i}{f_i=1}$ (Rahman et al. 2015). A schematic representation of the biological network is proposed in Fig. 1, representing the different components and how they interact with each other: plasmid carriers *P* can be converted to conjugation-able plasmid carriers *C* through gene expression, and the latter associate with a plasmid-free bacteria *F* to produce an exhausted donor *D* and a transconjugant *T* by conjugation. After a relaxation time, exhausted donors and transconjugants become plasmid carriers *P*. We included $f_T$ and $f_D$ in the model formulation to track quantitatively the amount of biomass having acquired a plasmid through conjugation. The fraction $f_C$ of plasmid carriers having expressed the *tra* genes allowing conjugation is included in the model to highlight the difference between conjugation and gene expression: gene expression is a nonlocal process affected by cells in the proximity of the plasmid carrier’s location, whereas conjugation is a local process requiring contact between the donor and the receptor cell.

The bulk liquid is considered as a homogeneous media, containing soluble substrates, that can be nutrients or toxic stressors such as metals or antibiotics. They are expressed in terms of concentration ${{\textbf {S}}} = (S_N,S_M,S_A)$, with $S_j(z,t), j\in J_S=\{N,M,A\}$. Soluble substrates are transported from the bulk to and through the biofilm by Fickian diffusion, where they react with biomass during bacterial metabolism, toxicity mechanisms and biosorption onto organic material.

The free boundary evolution is regulated by the metabolic growth of sessile bacterial species and the detachment into the bulk liquid. The detachment rate models the biomass loss due to surface erosion.

### Dynamics of Solid-Phase Species

The evolution of the biofilm components in space and time is modelled as an advective flux for the volume fraction of each component $f_i(z,t)$:

1 $$\begin{aligned} \frac{\partial f_i}{\partial t}+\frac{\partial }{\partial z}(uf_i)=r_{M,i}({{\textbf {f}}},{{\textbf {S}}}) + R_i(z,{{\textbf {f}}},{{\textbf {S}}}), \end{aligned}$$

with $(z,t) \in [0,L] \times (0,\infty )$ and $i\in J_f$, subject to suitable initial conditions. The velocity of the microbial mass displacement with respect to the substratum *u*(*z*, *t*) is obtained by summing Eq. 1 on *i*, recalling that $\sum _{i}{f_i=1}$:

2 $$\begin{aligned} \frac{\partial u}{\partial z}=\sum _{i\in J_f} r_{M,i} ({{\textbf {f}}},{{\textbf {S}}}), \ 0<z\le L(t), \ t>0. \end{aligned}$$

The first term on the right-hand side of Eq. 1 models the metabolic growth of active cells, consistently with previous works on continuum modelling of biofilms (Mattei et al. 2018). It depends on the fraction of said cells $f_i$, substrate concentration $S_N$ and growth inhibitor concentration such as metal $S_M$.

The function $R_i(z,{{\textbf {f}}},{{\textbf {S}}})$ represents all conversions of biomass from one state to another, through gene expression, conjugation and decay at position *z* and time *t* under the environmental conditions given by concentration of soluble substrates ${{\textbf {S}}}$ and biofilm fractions ${{\textbf {f}}}$. From mass balance considerations, we have $\sum _i R_{i} =0$, that is no conversion term participate in the expansion of the biofilm. Specifically, $R_i$ is defined as:

3 $$\begin{aligned} R_i(z,{{\textbf {f}}},{{\textbf {S}}})=D_i({{\textbf {f}}},{{\textbf {S}}})+C_i({{\textbf {f}}}) + G_i(z,{{\textbf {f}}},{{\textbf {S}}}). \end{aligned}$$

In Eq 3, the function $D_i({{\textbf {f}}},{{\textbf {S}}})$ models the decay of active species, i.e. their conversion from active to inert. The decay can be natural decay of cells or induced by antibiotics. The function $C_i({{\textbf {f}}})$ stands for all terms related to conjugation: the conversion from conjugation-able and plasmid-free into exhausted donors and transconjugants, which eventually relax into plasmid carriers. Finally, the function $G_i(z,{{\textbf {f}}},{{\textbf {S}}})$ models gene expression related processes, i.e. the conversion from plasmid carriers into conjugation-able and inversely. The latter function includes a non-local term to account for the dependence of recipient density around the donor and will be detailed in the following section.

#### Modelling Growth and Decay of Active Microbial Species

Based on previous modelling works (Ghasemi et al. 2018; D’Acunto et al. 2019) and experimental results (Newbury et al. 2022; Nanda et al. 2019; Baltrus 2013), we consider the following biological assumptions.

We consider bacteria growing at a rate that depends on the limiting substrate concentration through Monod kinetics. Moreover, the energy required for plasmid maintenance incurs a fitness cost for the bacteria, meaning that the growth rate of plasmid carriers is reduced by a factor related to the energy required for plasmid maintenance. Similarly, expressing the transfer genes allowing conjugation confers an even higher fitness cost, and so the growth rate of conjugation-able plasmid carriers is reduced by a factor related to the energetic cost of plasmid maintenance and additional gene expression. The fitness costs are quantified as constant parameters denoted as $\lambda _i$, which reduce the growth rate by the factor ($1 - \lambda _i$). This is consistent with the definition of fitness costs in the modelling (Leclerc et al. 2019) and experimental (Newbury et al. 2022) literature.

Metals affect bacterial growth by inhibition. Active biomass carrying plasmid are less sensitive to metal inhibition, and a correction factor accounts for this compensation by modifying the value of the inhibition constant. If the plasmid does not provide protective mechanisms from metals, the compensation constant value is fixed to 1.

EPS are produced during bacterial growth. We consider the EPS production of cells to be the same whether the bacteria carries a plasmid or not.

Bacterial decay can occur naturally or through the bactericidal action of antibiotics. Bacterial killing by antibiotics is modelled by a Hill toxicity function, with a correction coefficient to account for protection from plasmid carriage, when applicable. We consider that transconjugants and exhausted donors do not decay nor grow, as they model a transitory state.

Under the previous assumptions, we define the biomass conversion rates of fractions $f_F$, $f_P$, $f_C$ and $f_{EPS}$ as

4 $$\begin{aligned} r_{M,F}= &  (1-k_{EPS})\mu _{F} f_F, \ \mu _{F}=\mu _{max} \frac{S_N}{K_N + S_N}\frac{K_{I,M}}{K_{I,M}+S_M}, \end{aligned}$$

5 $$\begin{aligned} r_{M,P}= &  (1-k_{EPS})\mu _{P} f_P, \ \mu _{P}=\mu _{max}\frac{S_N}{K_N + S_N}\frac{\frac{K_{I,M}}{\chi _M}}{\frac{K_{I,M}}{\chi _M}+S_M}(1-\lambda _1), \end{aligned}$$

6 $$\begin{aligned} r_{M,C}= &  (1-k_{EPS})\mu _{C} f_C, \ \mu _{C}=\mu _{max}\frac{S_N}{K_N + S_N}\frac{\frac{K_{I,M}}{\chi _M}}{\frac{K_{I,M}}{\chi _M}+S_M}(1-\lambda _2), \end{aligned}$$

7 $$\begin{aligned} r_{M,EPS}= &  k_{EPS} (\mu _{F} f_F + \mu _{P} f_P + \mu _{C} f_C), \end{aligned}$$

where $\mu _{max}$ is the maximum growth rate, $k_{EPS}$ is the EPS fraction produced during growth, and $K_N$ is the half saturation constant associated with Monod bacterial growth. $K_{I,M}$ is the inhibition constant due to metal toxicity, corrected with the coefficient $\chi _M$ for plasmid carriers. $\lambda _1$ models the fitness cost of plasmid maintenance on plasmid-carrying bacteria $f_P$. $\lambda _2 \ge \lambda _1$ models the fitness cost of plasmid maintenance and additional gene expression on bacteria that bear a plasmid and express the *tra* gene $f_C$.

The functions modelling the decay of all active species into inert material are defined as follows:

8 $$\begin{aligned} D_F= &  - k_df_F - \kappa _{max}\frac{S_A^h}{MIC^h+S_A^h}f_F, \end{aligned}$$

9 $$\begin{aligned} D_P= &  - k_df_P - \chi _A \kappa _{max}\frac{S_A^h}{MIC^h+S_A^h}f_P, \end{aligned}$$

10 $$\begin{aligned} D_C= &  - k_df_C - \chi _A \kappa _{max}\frac{S_A^h}{MIC^h+S_A^h}f_C, \end{aligned}$$

11 $$\begin{aligned} D_I= &  k_d(f_F+f_P+f_C) + \kappa _{max}\frac{S_A^h}{MIC^h+S_A^h} (f_F + \chi _A f_P + \chi _A f_C), \end{aligned}$$

where $k_d$ is the natural decay constant, $\kappa _{max}$ is the maximum killing rate by antibiotic, *h* is the Hill coefficient of antibiotic, *MIC* is the minimum inhibitory concentration for antibiotic and $\chi _A$ is the correction coefficient to account for plasmid protection.

#### Modelling the Influence of Environmental Conditions on Gene Expression

The following biological assumptions are formulated based on experimental observations from the literature (Stalder and Top 2016; Bose and Grossman 2011; Singh et al. 2013; Lambertsen et al. 2004; Koraimann and Wagner 2014; Lin et al. 2019; Xu et al. 2022; Pallares-Vega et al. 2021).

The regulation of the *tra* gene is dependent on the social behavior displayed by the bacterial community, mainly due to its significant energetic cost. We assume that within the biofilm, the default state for the expression of the *tra* gene is OFF, and that under favorable environmental conditions, the donor will express them and thus become a transfer competent cell. Recipient-sensing is the first mechanism considered in this work that influences the *tra* gene expression: the more potential recipients around and the more likely the plasmid carrying cell is to become conjugation-able. Experimental studies have reported the existence of a negative auto-regulation mechanism: cells containing the conjugative element secrete an inhibitory peptide. Surrounding recipient cells can communicate their presence by reducing the concentration of this peptide when they possess an appropriate uptake system. The expression of the *tra* gene is turned ON during a burst, and then returns to the default OFF state. Gene expression is also dependent on the cellular fitness: the more cells are active at position *z* and time *t*, the more energy they have to dedicate to conjugation.

Additionally, metal stress has been abundantly proved to influence conjugation in bacterial populations. This effect is mainly attributed to a reactive oxygen species (ROS) generation, which triggers SOS response and alteration of the cell outer membrane, thus making plasmid transfer from a donor to a receptor cell easier. Here, we model the effect of metal selective pressure on gene expression by the promotion of the conjugative transfer of plasmids through oxidative stress caused by ROS generation. However, under too high metal concentrations, the promotion effect is hindered by the inhibition of cellular functions by metal stress.

The transition from ON to OFF state and inversely of the *tra* gene expression is modelled by the functions $G_P$ and $G_C$. They are defined as:

12 $$\begin{aligned} G_P= &  - r_{ON} + r_{OFF}, \end{aligned}$$

13 $$\begin{aligned} G_C= &  r_{ON} - r_{OFF}. \end{aligned}$$

Based on the previous assumption, we include the influence of recipient-sensing through a non-local term to account for the spatial dependence and define $r_{ON}$, a kinetic rate describing the *tra* gene expression and thus the ability of plasmid carrying cells to conjugate. This rate describes the conversion of the fraction $f_P$ into $f_C$:

14 $$\begin{aligned} r_{ON}= \nu _{ON} f_P \eta \frac{S_N}{S_N+K_N} \int _{0}^{L(t)}K(z-\zeta )f_F(\zeta ,t)d\zeta , \end{aligned}$$

where the Monod term quantifies the individual fitness of the donor bacteria depending on substrate availability, $K(z-\zeta )$ is the kernel function and describes the influence of space on the recipient sensing, $\nu _{ON}$ is the gene expression regulation rate and $f_F(\zeta ,t)$ is the plasmid-free fraction. The function $\eta$ describes the effects of metal on gene expression:

15 $$\begin{aligned} \eta =(1+\varepsilon S_M)e^{-\left( \frac{S_M}{m}\right) ^a}, \end{aligned}$$

where $\varepsilon$ is the metals promotion coefficient of gene expression, *a* and *m* are parameters related to gene expression inhibition by metals. This formulation was calibrated based on experimental data from Lin et al. (2019) and qualitatively reproduces different profiles found in the literature.

The kernel function was defined as an exponential function decreasing with $z- \zeta$, in order to account for the limitations of recipient-sensing:

16 $$\begin{aligned} K(z-\zeta )=e^{-k_{ON}(z-\zeta )^2}, \end{aligned}$$

where $k_{ON}$ is a scale parameter related to the recipient-sensing process, influencing the extension of the recipient-sensing mechanism.

We define $r_{OFF}$ as a first order kinetic modelling the return of conjugation-able bacteria to the default state:

17 $$\begin{aligned} r_{OFF}= \uptau _{burst}f_C, \end{aligned}$$

where $\uptau _{burst}$ is the kinetic rate associated with the duration of a burst in *tra* gene expression.

#### Modelling Conjugation

Based on previous modelling work of plasmid transmission by conjugation (Leclerc et al. 2019) and experimental observations from the literature (Wan et al. 2011), we formulate the following biological assumptions.

Conjugation is modelled as a mass action kinetic between conjugation-able plasmid carriers and plasmid free cells. This is consistent with most of the modelling works on conjugation in the literature.

Conjugation is energetically costly and produces an exhausted donor and a transconjugant, which are inactive for a given time before converting into plasmid carriers. We do not consider that plasmid carriers produced through conjugation have the *tra* gene expressed, as its default state is OFF.

We consider that all active cells within the biofilm are capable of conjugation and are compatible with plasmid-carriage. While this assumption does not exactly represent the complexity of bacterial biofilms ecology, we selected numerical values for conjugation parameters for pKJK5, a plasmid that carries resistance to antibiotics and is broad-range, i.e. is able to replicate and stably maintain the genes it carries in taxonomically distant species. As we consider only one plasmid, we also do not consider plasmid incompatibility groups, i.e. the presence of a plasmid in a host cell preventing the integration of another plasmid from a different incompatibility group.

Accordingly, we define the following rates related to conjugation:

18 $$\begin{aligned} C_F= &  - \gamma f_F f_C, \end{aligned}$$

19 $$\begin{aligned} C_P= &  \uptau _D f_D + \uptau _T f_T, \end{aligned}$$

20 $$\begin{aligned} C_C= &  - \gamma f_F f_C, \end{aligned}$$

21 $$\begin{aligned} C_D= &  \gamma f_F f_C - \uptau _D f_D, \end{aligned}$$

22 $$\begin{aligned} C_T= &  \gamma f_F f_C - \uptau _T f_T, \end{aligned}$$

where $\uptau _T,\uptau _D$ are the kinetic rates associated with the relaxation of transconjugants and exhausted donors, respectively and $\gamma$ is a kinetic constant related to conjugation.

### Dynamics of Soluble Substrates

The profile of soluble substrates is governed by the following set of parabolic partial differential equations:

23 $$\begin{aligned} \begin{aligned} \frac{\partial S_j}{\partial t}-\frac{\partial }{\partial z}\left( D_{S,j}\frac{\partial S_j}{\partial z}\right) =r_{S,j}({{\textbf {f}}},{{\textbf {S}}}),\ 0<z<L(t), \ t>0, \ j\in J_S, \end{aligned} \end{aligned}$$

where $D_{S,j}$ is the diffusivity coefficients of soluble substrate *j* within the biofilm. The reaction term $r_{S,j}$ corresponds to the conversion rate of substrate *j*, i.e. the rate at which it is assimilated, produced or immobilized by active microbial species.

We model the burden of plasmid maintenance into the fitness cost, as described above. Hence, we consider the yield associated to substrate conversion to be the same for $f_F$, $f_P$ and $f_C$.

We consider that metals are accumulated equally by all biomass, as the value of the metal accumulation rate taken from literature (Hill and Larsen 2005) corresponds to total metal accumulation in a biofilm without considering any difference between species, or between EPS, active and inactive bacteria. We do not consider the exhausted donors and transconjugants to accumulate metals, as they are transitory states.

Antibiotics are consumed during cell killing, at a rate proportional to the Hill toxicity described in Sect. 2.1.1. Additionally, antibiotics are subject to an abiotic decay, consistently with Ghasemi et al. (2018). Plasmid-borne resistance to antibiotic means that plasmid-carrying bacteria degrade less antibiotics than plasmid-free, as they interact less with antibiotics. The interactions between antibiotics and metals are neglected—i.e. synergistic effect on toxicity (Poole 2017) or reactions between metals and antibiotics.

Then, soluble substrates conversion rates are defined as:

24 $$\begin{aligned} r_{S,N}= &  -\frac{1}{Y_N}\rho \left( f_P \mu _{P} + f_F \mu _{F} + f_C \mu _{C}\right) , \end{aligned}$$

25 $$\begin{aligned} r_{S,M}= &  -\delta _M \left( f_F + f_P + f_C + f_{EPS} + f_I \right) ,\end{aligned}$$

26 $$\begin{aligned} r_{S,A}= &  -\frac{\kappa _{max}\rho }{Y_A}\frac{S_{A}^h}{MIC^h+S_{A}^h}(f_F + \chi _A f_C + \chi _A f_P) - \vartheta _A S_A, \end{aligned}$$

where $Y_N$ is the yield associated to substrate conversion and $Y_A$ is the antibiotic efficiency. $\delta _M$ is the metal accumulation rate by biofilm, and $\vartheta _A$ is the abiotic decay rate of antibiotic.

### Free Boundary Evolution

The free boundary evolution depends on the biofilm velocity and the detachment flux and is derived from the global mass balance on the biofilm domain [0, *L*(*t*)]:

27 $$\begin{aligned} {\dot{L}}(t)=u(L(t),t)-\sigma _d(L(t),t), \ t>0. \end{aligned}$$

Here, $\sigma _d$ is the detachment flux and models erosion at the interface between biofilm and bulk liquid. It is modelled as a deterministic process, through a continuous flux:

28 $$\begin{aligned} \sigma _d=\delta L^2, \end{aligned}$$

where $\delta$ is the detachment constant.

Notably, as specified in Sect. 2.1, the only terms contributing to the free boundary evolution are reaction terms related to metabolic growth, as all other terms are modelling a conversion of fractions and hence do not affect the biofilm velocity:

29 $$\begin{aligned} \sum _{i \in J_f} R_i(z,{{\textbf {f}}},{{\textbf {S}}})=0. \end{aligned}$$

### Initial and Boundary Conditions

Equation 1 is associated with the following initial conditions:

30 $$\begin{aligned} f_i(z,0)=f_{i,0}(z), \ i\in J_f, \ 0 \le z \le L_0, \end{aligned}$$

where $L_0$ is the biofilm initial thickness and $f_{i,0}$ is the initial volume fraction of solid phase component *i*.

The boundary condition for Eq. 2 is given by:

31 $$\begin{aligned} u(0,t)=0, \ t>0. \end{aligned}$$

which means that no biomass flux is assumed at the biofilm support $z=0$.

Initial-boundary conditions for Eq. 23 are set as:

32 $$\begin{aligned} &  S_j(z,0)=S_{j,0},\ j\in J_S, \end{aligned}$$

33 $$\begin{aligned} &  \frac{\partial S_j}{\partial z}(0,t)=0,\ S_j(L(t),t)=S_j^*(t), \ t>0, \ j\in J_S. \end{aligned}$$

The functions $S_{j,0}$ in Eq. 32 describe the initial concentrations of soluble substrates within the biofilm. The two boundary conditions stated in Eq. 33 correspond to a null flux at the substratum and to the assignment of the substrates concentrations value at the moving boundary $z=L(t)$, respectively. The functions $S_j^*$ describe the concentrations of soluble substrates within the bulk liquid.

## Qualitative Analysis

In this Section, we discuss the existence and uniqueness of the solution for the present free boundary value problem, under the following assumptions:

- Zero detachment velocity: $\sigma _d=0$.
- Substrate diffusion and conversion are much faster than biofilm growth, which leads to a quasi-steady-state dynamics for substrates (Gaebler and Eberl 2018):
  34 $$\begin{aligned} -\frac{\partial }{\partial z}\left( D_{S,j}\frac{\partial S_j}{\partial z}\right) = r_{S,j}({{\textbf {f}}},{{\textbf {S}}}),\ 0<z<L(t), \ t>0, \ j\in J_S. \end{aligned}$$

The integro-differential equations governing the transport and conversion of solid-phase fractions $f_i$ are rewritten here for convenience:

35 $$\begin{aligned} \begin{aligned} \frac{\partial f_i}{\partial t}+\frac{\partial }{\partial z}(uf_i)&=r_{M,i}(\textbf{f},\textbf{S})+C_{i}(\textbf{f})+D_{i}(\textbf{f},\textbf{S}) \\&\quad +\delta _i {\bar{\nu }}_{ON}(\textbf{S}) \int _{0}^{L(t)}K(z-\zeta )f_F(\zeta ,t)d\zeta - \delta _i r_{OFF}, \end{aligned} \end{aligned}$$

with

36 $$\begin{aligned} f_i(z,0)= &  f_{i,0}(z), \end{aligned}$$

37 $$\begin{aligned} {\bar{\nu }}_{ON}(\textbf{S})= &  \nu _{ON} f_P (1+\varepsilon S_M)e^{-\left( \frac{S_M}{a}\right) ^m} \frac{S_N}{S_N+K_N}, \ \delta _i = {\left\{ \begin{array}{ll} 1, & \text {for}\ i=C \\ -1, & \text {for}\ i=P \\ 0, & \text {otherwise} \\ \end{array}\right. }\nonumber \\ \end{aligned}$$

Now, let us define the characteristic-like curves as follows:

38 $$\begin{aligned} \frac{\partial c}{\partial t}(z_0,t) =u(c(z_0,t),t),\ \ c(z_0,0)=z_0, \ z_0 \in [0,L_0], \ t>0. \end{aligned}$$

Then, Eq. (35) in characteristic coordinates becomes

39 $$\begin{aligned} \frac{df_i}{dt}(c(z_0,t),t)= &  F_i(\textbf{f}(c(z_0,t),t),\textbf{S}(c(z_0,t),t)) + \delta _i {\bar{\nu }}_{ON}(\textbf{S}(c(z_0,t),t))\nonumber \\ &  \quad \times \int _{0}^{c(L_0,t)}K(c(z_0,t)-\zeta )f_F(\zeta ,t)d\zeta , \end{aligned}$$

where

40 $$\begin{aligned} F_i= r_{M,i}+C_i+D_i - \delta _i r_{OFF} -f_i G, \ G= \sum _{i\in J_f} r_{M,i}. \end{aligned}$$

Integrating from 0 to *t*:

41 $$\begin{aligned} f_i(c(z_0,t),t)= &  f_{i,0}(z_0) +\int _{0}^t F_i(\textbf{f}(c(z_0,\tau ),\tau ),\textbf{S}(c(z_0,\tau ),\tau ))d\tau \nonumber \\ &  + \int _{0}^t\! \delta _i {\bar{\nu }}_{ON}(\textbf{S}(c(z_0, \tau ),\tau )) d\tau \int _{0}^{c(L_0,\tau )}\!\!\! K(c(z_0, \tau ){-}\zeta )f_F(\zeta ,\tau )d\zeta ,\nonumber \\ \end{aligned}$$

42 $$\begin{aligned} f_i(c(z_0,t),t)= &  f_{i,0}(z_0) +\int _{0}^t F_i(\textbf{f}(c(z_0,\tau ),\tau ), \textbf{S}(c(z_0,\tau ),\tau ))d\tau \nonumber \\ &  + \int _{0}^t \delta _i {\bar{\nu }}_{ON}(\textbf{S}(c(z_0,\tau ),\tau )) d\tau \nonumber \\ &  \times \int _{0}^{L_0} K(c(z_0,\tau )-c(\zeta _0,\tau ))f_F(\zeta _0,\tau ) \frac{\partial c}{\partial \zeta _0} d\zeta _0. \end{aligned}$$

where the change of variables $\zeta =c(\zeta _0,\tau )$ has been considered.

The integral Eq. (42) is equivalent to the differential initial value problem (35), (36), as it satisfies the integro-differential Eq. (35) and the initial condition (36).

Suitable integral equations can also be derived for differential Eqs. (2), (34) and (38) (see Frunzo and Mattei 2017 for the full discussion). An existence and uniqueness result can be proven for all the unknowns in the class of continuous functions $C^0 ([0, \ L_0]\times [0, \ T])$, with

$$\begin{aligned} T=& \min \left\{ T_1,\frac{h_{f,i}}{2 M_i}, \frac{h_{c,1}}{L_0 M_{c,1}}, \frac{h_{c,2}}{M_{c,2}} \right\} , \ i\in J_f \end{aligned}$$

where $h_{f,i},h_{c,1},h_{c,2}$ are positive constants, while $M_{i}, M_{c,1}, M_{c,2}$ are the maximum values of the integrand functions. We provide the complete statement and proof of the Theorem based on fixed point considerations in the Appendix A.

## Numerical Results

The present model is here used to carry out numerical studies of biological interest. Specifically, we describe numerical methods and report the parameter values in Sect. 4.1. Then, two sets of numerical simulations are performed: Sect. 4.2 investigates the influence of the metal resistance coefficient at different metal concentrations on plasmid spread and persistence, and Sect. 4.3 describes how selective pressure from metals might influence the resistance of a mature biofilm to the minimum inhibitory concentration (MIC) of an antibiotic.

### Setting Up Numerical Simulations

#### Numerical Methods

The model presented in the previous section was integrated using an in-house Matlab code. The method of lines is applied to integrate the diffusion–reaction of substrates (Györi 1988), while the method of characteristics is used to track the evolution of the biofilm components (D’Acunto and Frunzo 2011; Friedman and Kao 2014). The biofilm domain has been discretized into a mesh with $N=100$ intervals. The total number of time instants has been set to $M=10^5 \cdot T$, where *T* represent the final time of simulation expressed in days. Specific care is taken for solving the integro-differential hyperbolic equations that regulate the dynamics of $f_P$ and $f_C$. For this, we use a highly accurate integral formula on the non-uniform characteristics mesh, such as reported in Mohanty (2012).

After defining the characteristic lines (Eq. 38), the solution region $[0,\ L(t)]\times [0,\ T]$ can be discretized as follows:

43 $$\begin{aligned} 0= &  t_0 \cdots<t_j<\cdots t_M=T, \ dt=\frac{T}{M}, \nonumber \\ 0= &  c^j_0 \cdots<c^j_k< \cdots c^j_{N-1}< L^j, \end{aligned}$$

where $c^j_k$ and $L^j$ are derived approximating Eqs. 38 and 27 by finite differences:

44 $$\begin{aligned} c^j_k= &  c^{j-1}_k + u^{j-1}_k dt, \ j=1,\ldots ,M, \ k=0,\ldots ,N-1, \nonumber \\ L^j= &  L^{j-1} + u^{j-1}_L dt - \sigma _{d}^{j-1} dt, \ j=1,\ldots ,M, \end{aligned}$$

where $$u^{j-1}_k = u(c^{j-1}_k,t^{j-1})$$, $$u^{j-1}_L = u(L^{j-1},t^{j-1})$$, and $$\sigma _{d}^{j-1}=\delta \ (L^{j-1})^2$$.

Note that the initial spatial discretization $c^{0}_k, \ k=1,\ldots ,N-1$ is assigned.

We define the variable mesh size:

45 $$\begin{aligned} h_{k+1}^j=c_{k+1}^j-c_{k}^j, \ k=0,\ldots ,N-1, \ j=0,\ldots ,M, \end{aligned}$$

where $h_{k+1}^j>0 \ \forall j,k$ due to the monotonicity of *u* with respect to the spatial coordinate.

The off-step points are defined by:

46 $$\begin{aligned} \begin{aligned} &  c_{k+\frac{1}{2}}^j = c_{k}^j+\frac{h_{k+1}^j}{2}, \ c_{k-\frac{1}{2}}^j =c_{k}^j-\frac{h_{k}^j}{2}, \\ &  \quad k=1,\ldots ,N-1, \ j=1,\ldots ,M. \end{aligned} \end{aligned}$$

For a specific time, the integral term is decomposed along the characteristics lines as follows:

47 $$\begin{aligned} \int _{0}^{L^j}K(c^j-\zeta )f_F^j(\zeta )d\zeta =\sum _{k=0}^{N} \int _{c^j_k}^{c^j_{k+1}}K(c^j-\zeta )f_F^j(\zeta )d\zeta . \end{aligned}$$

Following the result given by Evans (1993), we have:

48 $$\begin{aligned} \int _{c^j_k}^{c^j_{k+1}}K(c^j-\zeta )f_F^j(\zeta )d\zeta =\frac{h^j_{k+1}}{6}\left( \Phi ^j_k + 4\Phi ^j_{k+\frac{1}{2}}+\Phi ^j_{k+1}\right) , \ k=1,\ldots ,N,\nonumber \\ \end{aligned}$$

where $\Phi ^j_k$, $\Phi ^j_{k+\frac{1}{2}}$ and $\Phi ^j_{k+1}$ are defined as follows:

49 $$\begin{aligned} \Phi _k^j(c^j)= &  K\left( c^j-c^j_{k}\right) f_F^j\left( c^j_k\right) , \end{aligned}$$

50 $$\begin{aligned} \Phi ^j_{k+\frac{1}{2}}(c^j)= &  K\left( c^j-c^j_{k+\frac{1}{2}}\right) f_F^j(c^j_{k+\frac{1}{2}})=K\left( c^j-c^j_k {-}\frac{h^j_{k+1}}{2}\right) f_F\left( c^j_k{+}\frac{h^j_{k+1}}{2}\right) ,\nonumber \\ \end{aligned}$$

51 $$\begin{aligned} \Phi ^j_{k+1}(c^j)= &  K\left( c^j-c^j_{k+1}\right) f_F^j\left( c^j_{k+1}\right) =K\left( c^j{-}c^j_k-h^j_{k+1}\right) f_F^j\left( c^j_k{+}h^j_{k+1}\right) .\nonumber \\ \end{aligned}$$

Finally, by repeated application of Eq. 48 we obtain:

52 $$\begin{aligned} \int _{0}^{L^j}K(c^j-\zeta )f_F^j(\zeta )d\zeta =\sum _{k=0}^{N}\frac{h^j_{k+1}}{6}\left( \Phi ^j_k + 4\Phi ^j_{k+\frac{1}{2}}+\Phi ^j_{k+1}\right) . \end{aligned}$$

Equation 52 is implemented in the numerical code to integrate the integro-differential equations tracking the fractions of plasmid carriers and conjugation-able plasmid carriers.

#### Parameter Values

Numerical values for parameters associated with plasmid were taken from experimental works in the literature for plasmid pKJK5, a broad-range plasmid associated with antibiotic resistance. As all processes associated with plasmid carriage and conjugation are very specific and depend on the bacterial species and plasmid, this plasmid was chosen to be qualitatively representative of a conjugative resistance plasmid compatible with a large proportion of bacterial species. Mercury was chosen as the metal applied to the system as it can have an influence from very low levels due to its toxicity and based on effects on conjugation from Lin et al. (2019), as well as data availability on its accumulation by a microalgal biofilm (Hill and Larsen 2005). The diffusion coefficient of mercury in the biofilm was chosen in the typical range of metal diffusion (D’Acunto et al. 2019). All numerical simulations were run using parameters given in Table 1. All parameter values were taken, adapted or calibrated from the literature, except $\chi _M$, $\lambda _2$, $\nu _{ON}$, $k_{ON}$, that were introduced in this work. These parameters were chosen on the basis of the following considerations:

- Metal resistance coefficient $\chi _M$ was varied in a range from 0.1 to 1. These values correspond to the same resistance given to antibiotics to no resistance at all, respectively.
- Fitness cost of conjugation-able bacteria $\lambda _2$ was varied from 0.1 to 0.75. Biologically, this represents varying the value of the energetic cost of expressing the *tra* gene from none additional to plasmid maintenance to a significant burden of dedicating $75\%$ of cellular fitness towards it.
- Gene expression regulation coefficient $\nu _{ON}$ was chosen to have a gene expression allowing conjugation close to rates observed in the literature.
- Recipient-sensing coefficient $k_{ON}$ was chosen to have the kernel function reaching approximately zero at $z-\zeta = 25 \mu m$. Physically, it represents the limits of recipient-sensing within the biofilm bacterial population.

All parameter values used for model simulations are reported in Table 1, while initial and boundary values are provided in Table 2.

**Table 1:** Parameters used for model simulations

Parameter	Definition	Value	Unit	References
*Kinetic parameters*				
$\mu _{max}$	Maximum specific growth rate	6.0	d$^{-1}$	Eberl and Association (2006)
$K_{N}$	Half saturation constant for nutrient *N* (DOC)	4.0	g m$^{-3}$	Eberl and Association (2006)
$Y_{N}$	Yield bacteria on nutrient *N* (DOC)	0.63	*g*/*g*	Eberl and Association (2006)
$k_{d}$	Decay rate of bacteria	0.08	d$^{-1}$	Eberl and Association (2006)
$k_{EPS}$	*EPS* fraction produced during bacterial growth	0.18	–	Tenore et al. (2021)
*Physical parameters*				
$\rho$	Biofilm density	50000	g m$^{-3}$	Tenore et al. (2021)
$D_{N}$	Diffusion coefficient of nutrient *N* (DOC)	$8.3 \cdot 10^{-5}$	$\textrm{m}^2\textrm{d}^{-1}$	Russo et al. (2022)
$D_{M}$	Diffusion coefficient of metal *M*	$5 \cdot 10^{-5}$	$\textrm{m}^2\textrm{d}^{-1}$	(a)
$D_{A}$	Diffusion coefficient of antibiotic	$8.64 \cdot 10^{-6}$	$\textrm{m}^2\textrm{d}^{-1}$	Chambless et al. (2006)
$\delta$	Detachment coefficient	2000	$\textrm{m}^{-1}\textrm{d}^{-1}$	D’Acunto et al. (2021)
*Toxicity related parameters*				
$Y_A$	Antibiotic efficiency	375	$g(COD)/g(S_A)$	adapted from Ghasemi et al. (2018)
$\delta _{M}$	Metal accumulation rate by biofilm	0.0615	$\mathrm{g\,m}^{-3}\textrm{d}^{-1}$	Hill and Larsen (2005)
*h*	Exponent of Hill function for removal by antibiotic	2.5	–	Ghasemi et al. (2018)
*MIC*	Minimum inhibitory concentration for antibiotic	0.034	$\mathrm{g\,m}^{-3}$	Ghasemi et al. (2018)
$\chi _A$	Resistance coefficient of antibiotic	0.1	–	adapted from Ghasemi et al. (2018)
$\kappa _{max}$	Maximum killing rate by antibiotic	30	d$^{-1}$	Ghasemi et al. (2018)
$\theta _{A}$	Abiotic decay rate of antibiotic	0.001	d$^{-1}$	Ghasemi et al. (2018)
$K_{I,M}$	Inhibition constant by metal toxicity (Hg)	16.6	g m$^{-3}$	Lin et al. (2019)
$\chi _M$	Resistance coefficient of metal	0.1–1	–	This study
*Parameters related to conjugation and gene expression*				
$\lambda _1$	Fitness cost of plasmid carriage	0.1	–	Newbury et al. (2022)
$\lambda _2$	Fitness cost of expression of *tra* genes	0.1–0.75	–	This study
$\nu _{ON}$	Gene expression regulation rate	$1 \cdot 10^{4}$	$\textrm{m}^{-1}\textrm{d}^{-1}$	This study
$\gamma$	Conjugation rate constant	24	d$^{-1}$	Li et al. (2019)
$k_{ON}$	Gene expression regulation coefficient	$1 \cdot 10^{10}$	m$^{-2}$	This study
$\uptau _{burst}$	Gene expression burst rate	8	d$^{-1}$	Li et al. (2019)
$\uptau _{T}$	Transconjugants relaxation rate	16	d$^{-1}$	Wan et al. (2011)
$\uptau _{D}$	Exhausted donors relaxation rate	48	d$^{-1}$	Wan et al. (2011)
*a*	Gene expression inhibition factor	2.8	–	Lin et al. (2019)
*m*	Gene expression inhibition factor	1.5	$\textrm{g}\,\textrm{m}^{-3}$	Lin et al. (2019)
$\varepsilon$	Gene expression promotion factor	2.6	$\textrm{m}^{3}\,\textrm{g}^{-1}$	Lin et al. (2019)

(a) In the range of values present in the literature (Russo et al. 2023; Lide 2005)

**Table 2:** Initial and boundary values used for model simulations

Variable	Value	Unit
*Initial conditions*		
$L_{0}$	30	$\upmu \,\textrm{m}$
$f_{F,0}$	0.6	–
$f_{P,0}$	0.2	–
$f_{EPS,0}$	0.1	–
$f_{I,0}$	0.1	–
$f_{C,0}$	0	–
$f_{T,0}$	0	–
$f_{D,0}$	0	–
$S_{N,0}$	20	$\textrm{g}\,\textrm{m}^{-3}$
$S_{M,0}$	*varied*: $0-20$	$\textrm{g}\,\textrm{m}^{-3}$
$S_{A,0}$	0	$\textrm{g}\,\textrm{m}^{-3}$
*Boundary conditions*		
$S_N^*$	20	$\textrm{g}\,\textrm{m}^{-3}$
$S_M^*$	*varied*: 0–20	$\textrm{g}\,\textrm{m}^{-3}$
$S_A^*$	*varied*: 0–0.034	$\textrm{g}\,\textrm{m}^{-3}$

While plasmid-bearing can be beneficial to bacteria in given environmental conditions, the fitness effect is not entirely clear. For example, a plasmid giving resistance mechanism towards a toxic stressor can be beneficial when its bacterial host is exposed to the contaminant, but become detrimental when the host is not exposed to the stressor (Vogwill and MacLean 2015). The same authors also noted that the fitness cost associated with plasmid-borne resistance is generally less than the fitness cost associated with chromosomal resistance, thus explaining the high prevalence of plasmid-borne resistance. Moreover, the plasmid associated fitness cost is highly dependent on the plasmid-type and the bacterial species (Carroll and Wong 2018); and some authors even reported an increase in fitness when bearing a sulfonamide resistance-encoding plasmid in *E. coli* without selective pressure (Enne et al. 2004), underlining the potential difficulty of eliminating bacterial resistance carried by mobile genetic elements.

**Fig. 2:**
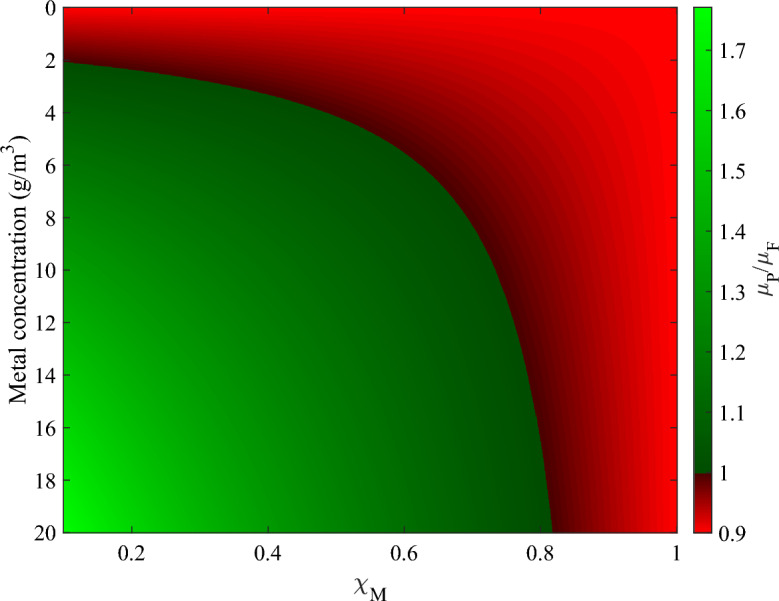
Ratio between $\mu _P$ and $\mu _F$. The MSW—Hg concentrations for which plasmid carriage is beneficial for the individual fitness of bacteria—depends on the value of the resistance coefficient and is highlighted in green: $\mu _P/\mu _F>1$ (Color figure online)

Parameter values were chosen to reflect behaviours observed experimentally, as highlighted in model presentation. One real-life behaviour that the model is able to reproduce is the existence of the Mutant Selection Window (MSW): the concentration range of the toxic stressor in which the growth rate of mutants is higher than the growth rate of plasmid-free bacteria. Consequently, the significant dissemination of plasmids is dependent upon the MSW (Trubenová et al. 2022). In Fig. 2, the ratio of the growth rate of plasmid-carrying and plasmid-free bacteria is presented. The area in green represents the domain of metal concentration and resistance coefficient for which plasmid carriers are advantaged as compared to plasmid-free cells. Practically, it corresponds to the area where Hg concentration and resistance coefficient are able to compensate the fitness cost of plasmid carriage. The line in black shows the parameter space for which their growth rate are identical, hence where plasmid carriers can still persist because they are competitive enough to coexist with plasmid-free cells. In red, plasmid free are advantaged and plasmid-carrying cells are at risk of being phased out from the biofilm. The MSW is the zone highlighted in green, according to the model formulation and the selected parameter values.

From this figure, it is evident that regardless of the resistance coefficient, the growth of plasmid carriers is consistently hindered when exposed to Hg concentrations below $2\,\textrm{g}\,\textrm{m}^{-3}$. Likewise, plasmid carrying bacteria are disadvantaged for all Hg concentrations when exhibiting low resistance, characterized by a resistance coefficient $\chi _M>0.82$. However, it is worth noting that this figure does not include conjugation, which is a dynamic process. At low Hg concentrations, especially at $\,\textrm{g}\,\textrm{m}^{-3}$ where gene expression is most promoted, the consequent increase in gene expression and hence $f_C$ can lead to more conjugation, which could enlarge the MSW and thus promote the persistence of plasmids in the biofilm.

Notably, metal within the biofilm is subject to a concentration gradient induced by diffusion limits and accumulation by biomass. The gradient might induce the emergence of distinct subpopulations within the biofilm, due to different environmental conditions.

### Influence of Selective Pressure on Plasmid Persistence

The objective of these numerical simulations is to assess the impact of selective pressure on plasmid persistence. We explore a broad spectrum of Hg concentrations, ranging from zero to levels that entirely impede conjugation. This also influences growth inhibition, as it varies among cells based on whether they harbor a plasmid or not. The selective pressure hence has an effect on plasmid spread through HGT events (transfer of plasmids through conjugation) and VGT (plasmid replication in daughter cells during cell division). Furthermore, we vary the resistance coefficient to evaluate its effect on the growth rate of plasmid carriers, and thus on the VGT process. We consider a biofilm growing from initial conditions reported in Table 2, exposed to concentrations of Hg from 0 to $20 \ \upmu \,\textrm{g}\,\textrm{m}^{-3}$. Antibiotic concentration at the boundary $S_A^*$ is set to zero.

In total, 40 simulations were conducted to investigate plasmid spread and persistence under different values of $\chi _M$, which ranged from 0.1 (representing a 10-fold increase in the inhibition constant when the cell carries a plasmid) to 1, indicating no resistance provided by plasmid carriage. Parameter variation and subsequent inhibition of cell growth is reported in Table 3.

**Table 3:** Parameter variations and influence on effective inhibition of plasmid-free cells and plasmid-carrying cells

$S_{M}^* (\textrm{g}\,\textrm{m}^{-3})$	Effective inhibition of $f_F$ (%)	Effective inhibition of $f_P$, for following values of $\chi _M$				
		0.1 (%)	0.25 (%)	0.5 (%)	0.75 (%)	1 (%)
0	0	0	0	0	0	0
1	5.7	0.6	1.5	2.9	4.3	5.7
2	10.8	1.2	2.9	5.7	8.3	10.8
3.5	17.4	2.1	5.0	9.5	13.7	17.4
5	23.1	2.9	7.0	13.1	18.4	23.1
10	37.6	5.7	13.1	23.1	31.1	37.6
15	47.5	8.3	18.4	31.1	40.4	47.5
20	54.6	10.8	23.1	37.6	47.5	54.6

**Fig. 3:**
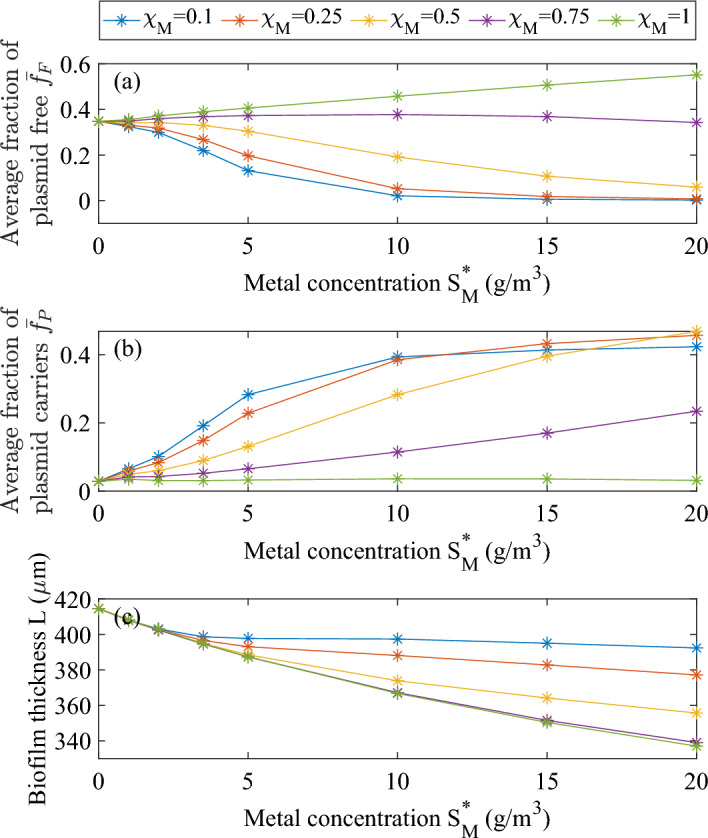
Average fraction of plasmid-free cells (**a**), plasmid-carrying cells (**b**) and biofilm thickness (**c**) after 100 days of simulation time, for different Hg concentrations in the bulk liquid $S_M^*$ and resistance coefficient values $\chi _M$. No antibiotics in the system: $S_A^*=0$ (color figure online)

In Fig. 3 fractions of plasmid-free, plasmid carrying bacteria and biofilm thickness are presented for different Hg concentrations and resistance coefficients. The higher the protection conferred by the plasmid is, and the less the biofilm thickness is affected by increasing Hg concentrations: plasmid-carrying cells are less inhibited and can keep growing, as can be seen in Fig. 3b. Oppositely, this is associated with a concentration of plasmid-free cells tending to zero for increasing Hg concentration (Fig. 3a).

One interesting observation is that when the metal concentration is $S_M=20\,\textrm{g}\,\textrm{m}^{-3}$, and the resistance coefficients are set to 0.25 and 0.5, the fraction of cells carrying the plasmid after 100 days is greater compared to the value obtained for $\chi _M=0.1$, despite a lower protection. Indeed, a lower resistance coefficient fosters a higher growth rate of plasmid-carrying cells, resulting in thicker biofilm. In thicker biofilms, diffusion limitations lead to stronger substrate gradients, and more intense decay in the inner regions. The result is a higher overall fraction of inert material $f_I$ and a lower fraction of plasmid-carrying cells $f_P$. Specifically, as the resistance coefficient decreases, the average inert fraction within the biofilm increases to 0.29, 0.36, and 0.40 for $\chi _M=0.5, \ 0.25, \ 0.1$, respectively (data not shown).

**Fig. 4:**
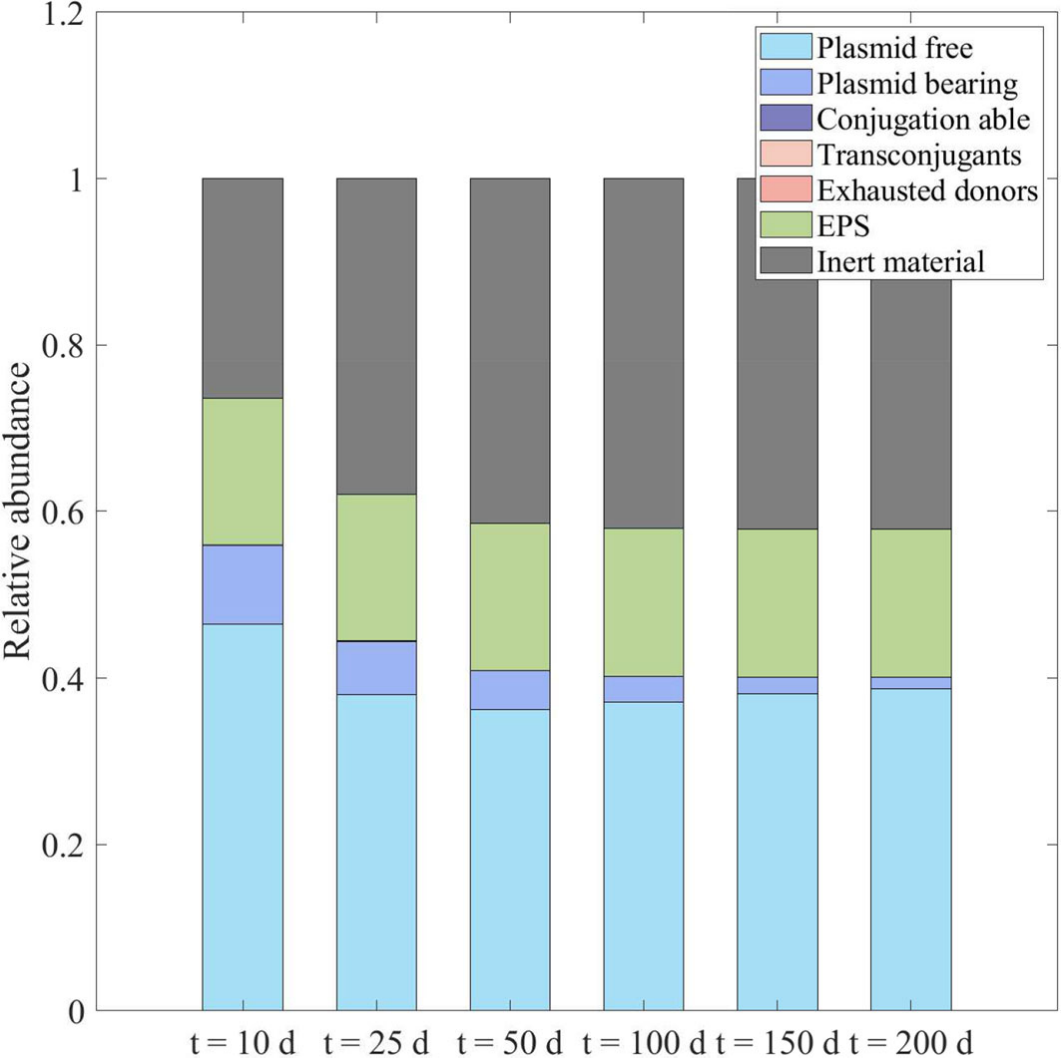
Solid-phase fractions within the biofilm at different times, for $S_M^* =2\,\textrm{g}\,\textrm{m}^{-3}$ and $\chi _M =1$. No antibiotics in the system: $S_A^*=0$ (color figure online)

Similarly, if the plasmid does not confer resistance benefits (i.e., $\chi _M = 1$), the fraction of cells carrying the plasmid after 100 days is not affected by the concentration of Hg. This is because both plasmid-carrying and plasmid-free cells are inhibited to the same degree. In such cases, the fitness cost associated with plasmid maintenance $\lambda _1$ disadvantages the fraction of plasmid-carrying cells $f_P$, leading to their eventual disappearance from the active population of the biofilm in longer simulations (trend shown in Fig. 4 for 200-day simulations at a Hg concentration of $2\,\textrm{g}\,\textrm{m}^{-3}$). With increasing Hg concentrations, there is a higher fraction of plasmid-free cells $f_F$ after 100 days, owing to a thinner biofilm and less substrate limitation despite higher inhibition. Notably, the fractions $f_C$, $f_T$ and $f_D$ do not accumulate in the biofilm as they are just transitory states, representing a burst in expression of the *tra* gene or a state of exhaustion after receiving or transmitting a plasmid, respectively.

**Fig. 5:**
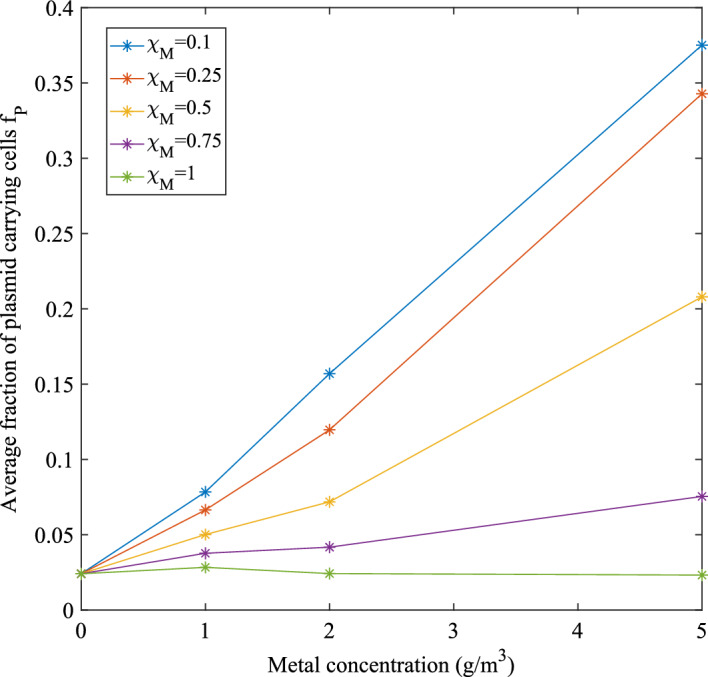
Average fraction of plasmid-carrying cells after 200 days of simulation time, for different Hg concentrations and resistance coefficient values. No antibiotics in the system: $S_A^*=0$ (color figure online)

Focusing on metal concentrations with an inhibition lower than $20\%$ on plasmid-free cells (i.e. a Hg concentration less than $5\ \textrm{g}\,\textrm{m}^{-3}$) allows to clearly see the influence of conjugation on plasmid persistence. As can be seen in Fig. 5, for a concentration of Hg equal to $1\ \textrm{g}\,\textrm{m}^{-3}$, the fraction of plasmid carrying bacteria has a slight increase, due to the promotion of conjugation at this concentration (Eq. 15). This effect attenuates at lower resistance coefficients, as vertical gene transfer is quantitatively more significant than conjugation. In this range of concentrations, the decrease in thickness due to increasing Hg concentration is close to linear, with a limited influence of the resistance coefficient. It is important to note that for Hg concentrations above $5\ \textrm{g}\,\textrm{m}^{-3}$, conjugation is negligible due to the very low value of $\eta (M)$, modelling the interference of Hg with cellular protein functionality. Therefore, the difference in plasmid persistence in this concentration range is solely due to the competition between plasmid-free and plasmid-carrying cells for limited space and nutrients within the biofilm domain, as they have different growth rates due to different inhibition kinetics and fitness cost (Table 3).

In this study, the fitness cost coefficient for plasmid maintenance $\lambda _1$ is set to 0.1 based on experimental data from plasmid pKJK5 (Newbury et al. 2022) and other modelling studies (Arya et al. 2021). A series of simulations were conducted to examine the impact of the fitness cost $\lambda _2$, which is associated with gene expression required for conjugation and varied it from 0.1 to 0.75. Interestingly, the results indicate that this parameter has very little influence on the VGT process. This is because the contribution to the biofilm growth of conjugation-able bacteria is negligible compared to plasmid-free and plasmid carriers, due to their low proportion within the biofilm. Thus, the primary effect of the fraction $f_C$ in this model formulation is to enable plasmid spread through HGT.

**Fig. 6:**
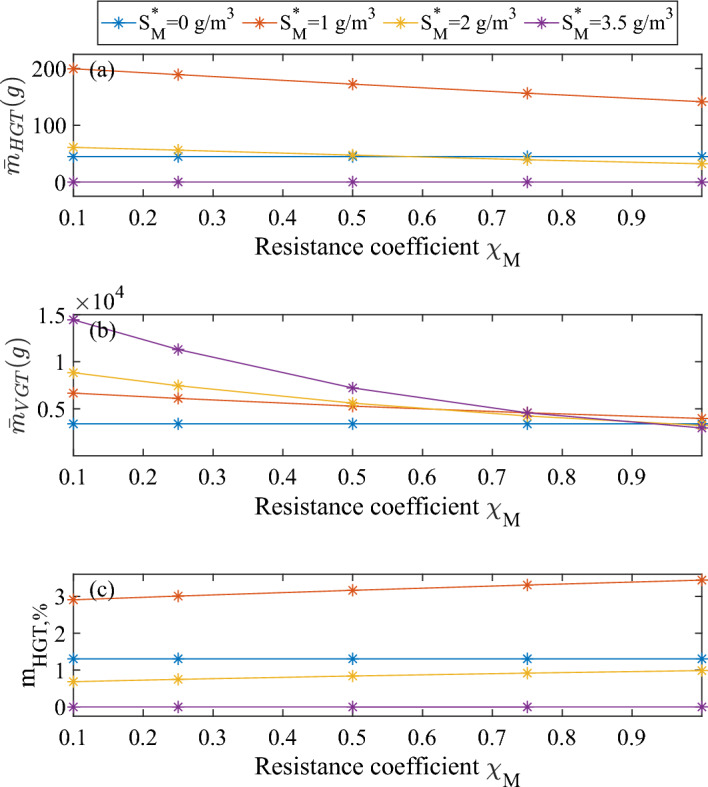
Total mass of plasmid spread contribution via HGT (**a**) or VGT (**b**) and proportion of HGT to the total plasmid dissemination (**c**), after 100 simulation days, for different metal concentrations and values of $\chi _M$. No antibiotics in the system: $S_A^*=0$. Biofilm surface area: $1 \ \textrm{m}^2$ (color figure online)

In Fig. 6, we aim to illustrate the contribution of HGT and VGT to plasmid spread. To this end, we plot three different indicators and compare them for different metal concentrations and resistance coefficients:

- The total mass of transconjugants generated, which quantifies the amount of cells having received a plasmid through conjugation - the form of HGT we model here: ${\bar{m}}_{HGT}= A \int _{0}^{t}d\tau \int _{0}^{L(\tau )}\rho \uptau _T f_Tdz$.
- The total mass of $f_P$, measuring the amount of plasmid disseminated through cell division of plasmid carriers: ${\bar{m}}_{VGT}= A \int _{0}^{t}d\tau \int _{0}^{L(\tau )}\rho r_{M,P}dz$.
- The proportion of HGT to the total plasmid dissemination: ${\bar{m}}_{HGT,\%} = {\bar{m}}_{HGT} / ({\bar{m}}_{HGT} + {\bar{m}}_{VGT})$.

These values have been evaluated within the biofilm after 100 days.

First, it is important to emphasize that HGT and VGT contributions cannot be completely separated from each other, as they are inherently interrelated. Indeed, the fraction of plasmid-carrying bacteria produced by HGT will influence the subsequent VGT and vice versa. So even if the HGT process might seem quantitatively negligible (Fig. 6c), in the end the plasmids produced through HGT allow a more consequent plasmid spread through VGT. This is also the reason why the HGT process, even at metal concentration of $1 \ \textrm{g}\,\textrm{m}^{-3}$ loses in intensity for increasing resistance coefficient: it comes from the decrease in VGT, due to the fact that plasmid carriers are more and more disadvantaged compared to plasmid-free cells at increasing resistance coefficient, leading to a drop in their growth rate and hence reduced vertical gene transfer. Naturally, this observation does not apply in the case of null metal concentration, for which the resistance coefficient has no effect at all on $\mu _P$. Finally, it is interesting to note that the contribution to new plasmid generation through HGT does not change significantly with the resistance coefficient (Fig. 6c): HGT and VGT are so interconnected that this ratio remains almost constant for varying $\chi _M$. However, the relative contribution to plasmid spread via horizontal gene transfer varies with the metal contribution due to the promotion/inhibition function $\eta (M)$.

**Fig. 7:**
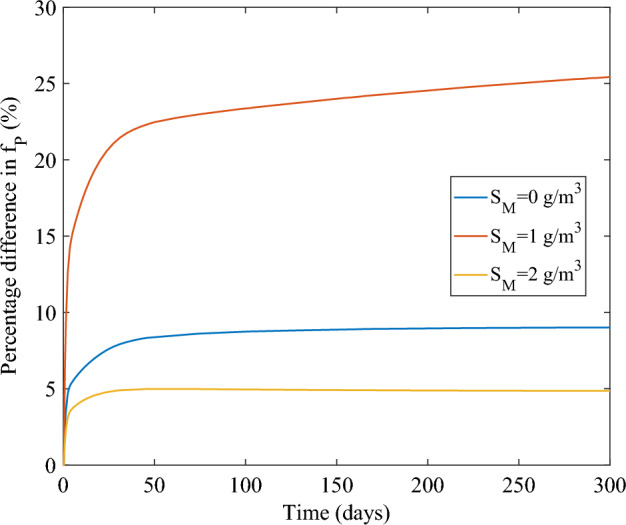
Percentage difference between plasmid carrying population with and without conjugation, for different metal concentrations (color figure online)

The close connection between HGT and VGT can be observed in Fig. 7. In this figure, we report the percentage difference between plasmid carrying population with and without conjugation, for different metal concentrations. This difference ranges from 5 to 26%, which is significantly higher than the proportion of HGT to total plasmid dissemination (Fig. 6c). This discrepancy can be attributed to the intricate relationship between these two processes: cells produced through HGT at a low rate subsequently proliferate and contribute significantly to plasmid dissemination through VGT. This leads to the observed higher differences in the plasmid-carrying population.

These findings align with experimental data regarding the proliferation of plasmid-mediated antimicrobial resistance under metal contamination (Chen et al. 2019; Zhang et al. 2018; Cai et al. 2023; Suzuki et al. 2012; Sun et al. 2021). Even without considering incompatibility groups for plasmid transfer and specificity of the transfer, the study of a broad-range plasmid allows to predict general results at the population scale within a growing biofilm. When quantitatively comparing horizontal and vertical gene transfer, the model accurately predicts that plasmid dissemination is predominantly influenced by vertical gene transfer. This is especially true at high metal concentrations, where conjugation is inhibited but not necessary for plasmid spread; indeed, in this range of concentration plasmid carriers have a huge growth advantage over plasmid-free cells and this is sufficient for plasmid persistence within the biofilm. However, conjugation becomes really relevant at low metal concentrations because it can extend the presence of plasmids within the bacterial population even in the absence of selective pressure, making it a complementary process to vertical gene transfer. Specifically, at low metal inhibition levels and thus limited influence of VGT on plasmid persistence, the promotion of conjugation enables the biofilm to sustain a resistant sub-population, consistently with experimental findings (Baker-Austin et al. 2006; Zhang et al. 2018). The fitness cost related to gene expression is neglectable in this model formulation, but the scientific community still lacks comprehensive knowledge about the fitness cost of gene expression and its impact on the VGT of plasmids to be able to conclude on the accuracy of this result. In this model formulation, the fact that $f_C$ is a transitory state allowing conjugation but not accumulating means that its contribution to growth will be neglectable. However, its production is a necessary step to conjugation and including the gene expression as a previous step to conjugation allowed to integrate two main aspects into the model:

- the non-locality of gene expression. Indeed, conjugation is a local process that requires the simultaneous presence of both donor and receptor cells at the position *z* to happen. On the other hand, recipient-sensing mechanisms make gene expression dependent on other state variables at $(z-\zeta )$ to happen at the position *z*.
- the fact that gene expression occurs as a burst phenomenon (Koraimann and Wagner 2014), with a kinetic rate allowing the return to the default state, without the *tra* genes expressed and its associated fitness cost.

### Impact of Metal and Antibiotic Co-resistance on Plasmid Persistence

As previously mentioned, the increasing prevalence of antibiotic resistance is a significant global health issue, largely resulting from the widespread use of antibiotics. The selective pressure exerted by metals can also have an impact on the spread of ARGs. Therefore, we are examining here how varying concentrations of a metal affects the biofilm resistance to antibiotics in two distinct scenarios: co-resistance ($\chi _m < 1$), where metals can affect both horizontal and vertical gene transfer, and promotion of conjugation of antibiotic resistance plasmids that do not confer resistance to the metal.

**Fig. 8:**
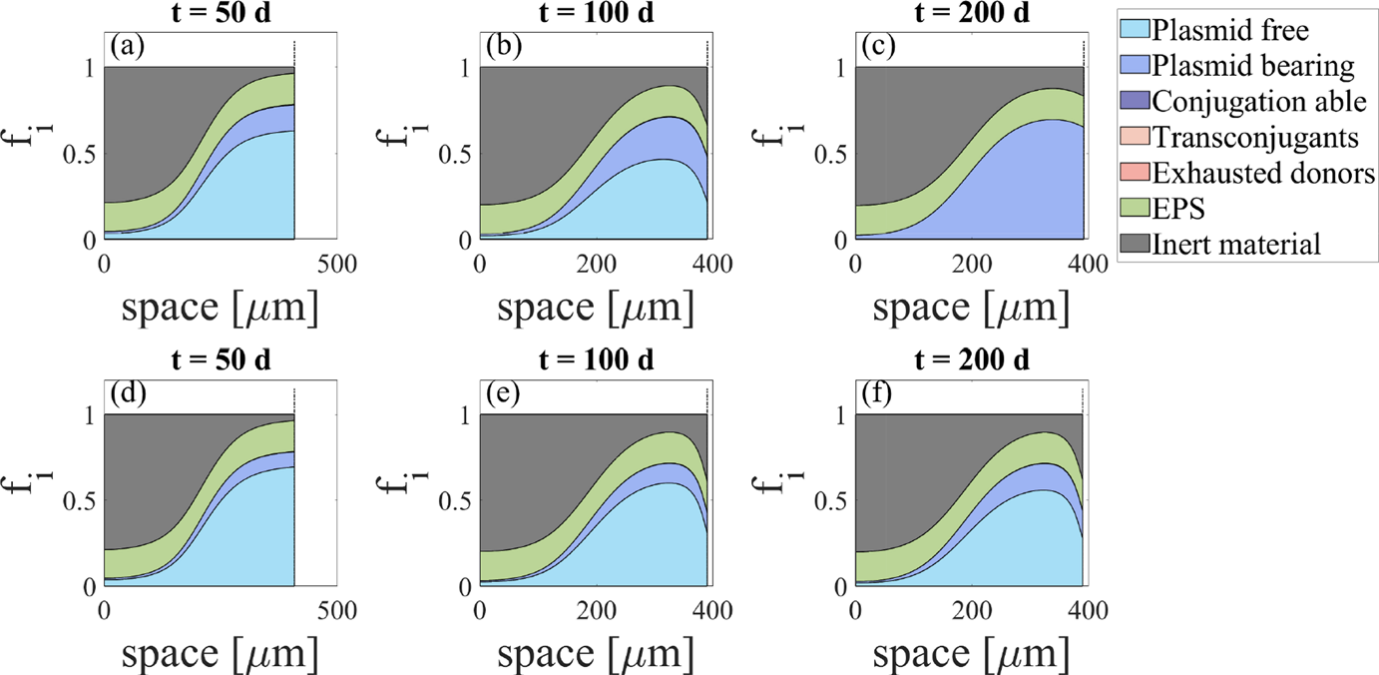
Volume fractions distribution within the biofilm at $t=50$ days (**a** and **d**), $t=100$ days (**b** and **e**) and $t=200$ days (**c** and **f**) for $\chi _M=0.1$ (**a**, **b** and **c**) and $\chi _M=1$ (**d**, **e** and **f**). Biofilm is exposed to antibiotics from $t=50$ days: $S_A^*=0.034 \ \textrm{g}\,\textrm{m}^{-3}$ (color figure online)

Antibiotics kinetics (Eqs. 8, 9, 10, 11, 26) were taken from Ghasemi et al. (2018) as Hill toxicity, and the value of $\chi _A$, the resistance coefficient, was selected equal to 0.1. Biologically, it means that the decay due to antibiotics kinetics of plasmid carrying bacteria is $90\%$ lower than for plasmid-free, due to the protection mechanisms. We consider the case of a growing biofilm from initial thickness of $30\,\upmu \,\textrm{m}$, exposed to low concentrations of Hg (0 to $5 \ \textrm{g}\,\textrm{m}^{-3}$). From $t=50$ days, the biofilm is exposed to an antibiotic concentration of $0.034 \ \textrm{g}\,\textrm{m}^{-3}$, corresponding to the MIC. Figure 8 compares the distributions of volume fractions within the biofilm for the case of $S_M^*=1 \ \textrm{g}\,\textrm{m}^{-3}$ and $\chi _M=0.1$—meaning co-resistance to Hg and antibiotic—to $S_M^*=1 \ \textrm{g}\,\textrm{m}^{-3}$ and $\chi _M=1$—meaning no resistance to Hg given by the plasmid.

**Fig. 9:**
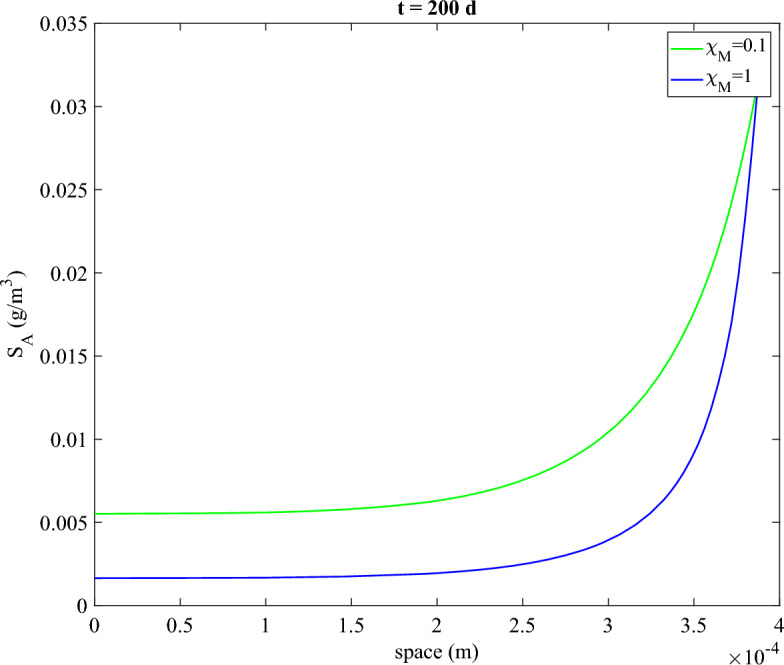
Antibiotic concentration within the biofilm after 200 days, for $\chi _M=0.1$ and $\chi _M=1$. Biofilm is exposed to antibiotics from $t=50$ days: $S_A^*=0.034 \ \textrm{g}\,\textrm{m}^{-3}$ (color figure online)

After 50 days, there is a small difference in the fraction of plasmid-carrying bacteria, as observed in the previous section. This difference increases drastically after 100 days, because of the additional influence of antibiotics applied at the MIC, significantly affecting the vertical gene transfer by promoting the plasmid-carrying bacteria. In the case of $\chi _M=0.1$, plasmid-free cells completely disappear from the biofilm, as they cannot compete with the ecological advantage given by the plasmid resistance to both antibiotic and Hg. In contrast, when $\chi _M=1$, the plasmid-free cells are still predominant in the biofilm despite the presence of antibiotics at the MIC. This highlights the crucial role of co-resistance in the mechanisms by which metals promote the persistence of plasmids at these concentrations. It is worth noting that plasmid carriers are predominantly found in the outer layer of the biofilm. This is likely due to limited diffusion of the antibiotic (as shown in Fig. 9), which makes selection of plasmid carriers more quantitatively significant in this region of the biofilm. Conversely, the metal concentration has a negligible influence on microbial spatial distribution, as metal gradients are not highly pronounced due to the low rate of bioaccumulation within the biofilm (data not shown). Additionally, the growth rate is higher in the outer layers due to the nutrient gradient, which also contributes to the higher prevalence of plasmid carriers in this region as the gene expression rate depends on nutrient availability (Eq. 14).

In the case of co-resistance, the antibiotic concentration reaching the inner part of the biofilm is 3 times higher compared to the scenario where the plasmid only carries antibiotic resistance (i.e., $\chi _M=1$). This comes from a lower antibiotic consumption, due to the prevalence of plasmid carrying bacteria from the selective pressure of Hg on the VGT process. As plasmid carriers with co-resistance are less affected by antibiotics, the reaction term associated with antibiotic consumption during cell killing (Eq. 26) is lower, allowing for a deeper penetration of the antibiotic within the biofilm. It is also worth noticing that the biofilm has a very slight difference in thickness, due to a lower protection in the case $\chi _M=1$ and, consequently, an insignificant difference in biofilm growth.

**Fig. 10:**
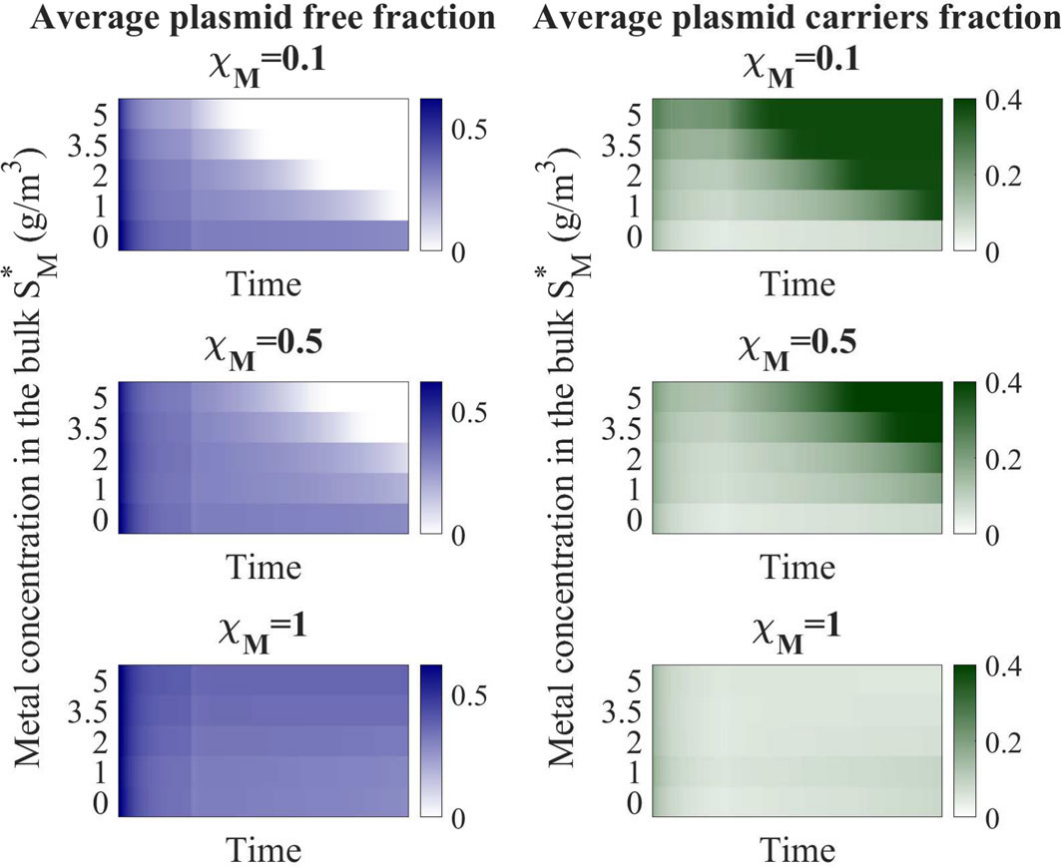
Plasmid free (blue) and plasmid carrying (green) fraction during 200 days of simulation time, for a biofilm growing under different Hg concentrations, for different resistance properties against metals $\chi _M$, and exposed to antibiotics from $t=50$ days: $S_A^*=0.034 \ \textrm{g}\,\textrm{m}^{-3}$. ${\bar{f}}_F$ is the average plasmid free fraction. ${\bar{f}}_P$ is the average plasmid carrying fraction (Color figure online)

Fig 10 presents the plasmid free, plasmid carrying after 200 days of simulations, for biofilms exposed to a selective pressure of Hg at concentrations from 0 to $5 \ \textrm{g}\,\textrm{m}^{-3}$.

The variation in the fraction of inert and EPS was found to be very small in the simulations and was not significant in the context of selective pressure after antibiotic injection (data not shown). Instead, the main factor affected by selective pressure is the $f_F:f_P$ ratio. This can be explained by the fact that the antibiotic-induced decay mainly occurs in the outer layers of the biofilm, where there is little to no accumulation of inert due to high biomass metabolic activity. Conversely, in the inner region of the biofilm, antibiotic penetration is limited by diffusion and $S_A$ is in low levels (Fig. 9). Hence, the decay process in this region of the biofilm is mainly the natural one and does not depend on whether the cells carry a resistance plasmid or not. As shown in Fig. 10, the resistance coefficient has a significant impact on the competition between plasmid-free and plasmid-carrying cells in the biofilm. The lower the resistance coefficient (that is the higher resistance of plasmid-carrying cells to metal), the sooner plasmid-free are phased out of the biofilm due to not being able to compete with plasmid-carriers. Additionally, for lower resistance coefficients plasmid-free phase out from the biofilm happens at lower Hg concentration. The case $\chi _M=1$ really shows the influence of HGT on the process, as no resistance to Hg is given by plasmid carriage: plasmid carrying cells are selected based on antibiotic concentration, but the only factor influencing the different results is Hg concentration and its influence on conjugation. $f_P$ are able to sustain a low fraction, especially at the optimum Hg concentration for promoting conjugation ($S_M^*=1\,\textrm{g}\,\textrm{m}^{-3}$).

The case $S_M^*=0$ gives same results for all three values of resistance coefficient, but the comparison of those results with higher Hg concentrations shows the relevance of selective pressure on plasmid persistence in the biofilm. These results also highlight the importance of co-resistance (or cross-resistance) to metals and antibiotics for the maintenance of ARG in biofilms.

**Fig. 11:**
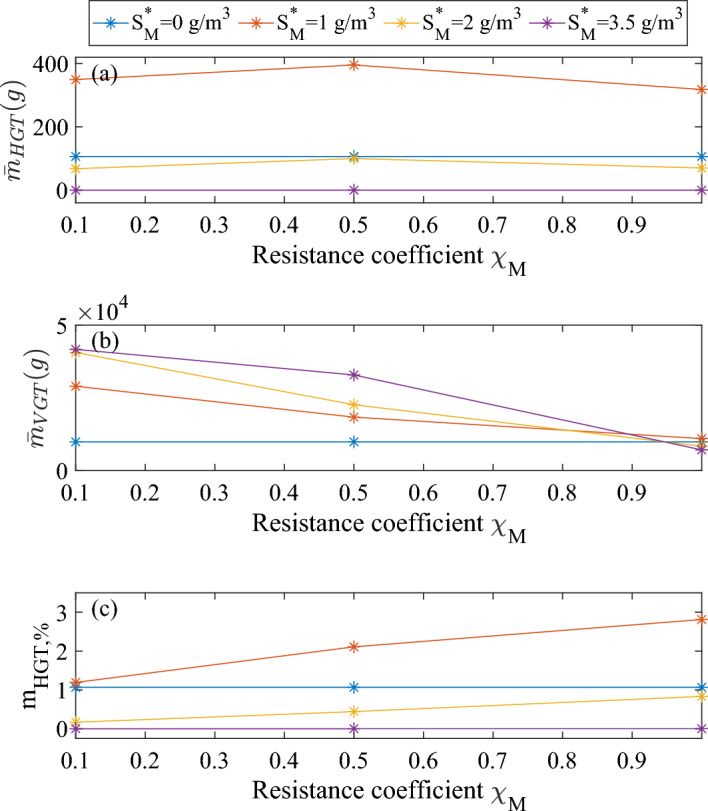
Total mass of plasmid spread contribution via HGT (**a**) or VGT (**b**) and proportion of HGT to the total plasmid dissemination (**c**), after 200 simulation days, for different metal concentrations and values of $\chi _M$. Biofilm is exposed to antibiotics from $t=50$ days: $S_A^*=0.034 \ \textrm{g}\,\textrm{m}^{-3}$. Biofilm surface area: $1 \ m^2$ (color figure online)

Figure 11 illustrates the cumulative plasmid spread over 200 days of simulation, for different values of metal concentrations and resistance coefficients. The difference between data displayed in this figure and Fig. 6 is the addition of the antibiotic with the Hill kinetics, which causes antibiotic-induced decay in addition to the natural decay (Eq. 11). Consequently, plasmid-carriers are much less disadvantaged than in the previous set of simulations, as the plasmid carriage provides protection against antibiotic action in all scenarios considered. It allows to point out a different limitation to horizontal plasmid transfer: the lack of surrounding receptor cells. Indeed, the trend for the HGT intensity does not decrease as in Fig. 6 for increasing $\chi _M$, but has a maximum for $\chi _M=0.5$. This deviation can be attributed to the fact that, for $\chi _M=0.1$, HGT is limited by the scarcity of receptor cells. Indeed, the production of $f_C$ through gene expression depends on receptor-sensing (Eq. 14) and hence on plasmid-free cells fraction. As they are phased out from the biofilm from t=195 days (data not shown) and in a low concentration before that, it results in a low value of $r_{ON}$ and consequently low conjugation rates. In this case, $\chi _M=0.5$ is the value of the resistance coefficient that allows the best ratio between donors and receptors for maximised conjugation, among the values simulated. For the same reason, the contribution of HGT in total plasmid spread increases for increasing resistance coefficient, in contrast to the nearly constant value observed in Fig. 6.

The numerical results from these simulations effectively replicate the co-resistance mechanisms outlined in the introduction. The increase in horizontal gene transfer at sub-inhibitory metal concentrations stems from an increase in the conjugation-able fraction of the plasmid carriers, allowing for a more consequent conjugation flux. The HGT contribution to plasmid dissemination, although it might seem negligible, has a significant impact on plasmid persistence as HGT and VGT are strongly inter dependent. However, conditions that are excessively favorable to plasmid-carrying cells can lead to the complete phasing out of plasmid-free cells, which will in turn result in no conjugation occurring since there are no available receptor cells. This outcome is a consequence of assuming that plasmids can be transferred to all active cells within the biofilm. Including cells incapable of receiving plasmids through conjugation in the model would not affect the overall result, as there would still be no receptor cells available.

The potential threat to human health via the promoted dissemination of ARG in the case of cross-resistance or co-resistance is outlined. Notably, even in the absence of antibiotic exposure, the co-resistance to a metal (in this case, Hg) promotes plasmid persistence and hence community resistance to antibiotics (Cai et al. 2023), increasing the chance of this ARG to be passed on to a human pathogen. The result of this phenomenon on the extended-term dynamics of the biofilm is an increased proportion of resistant bacteria, as previously documented in scientific literature (Xu et al. 2022; Baker-Austin et al. 2006).

## Conclusions and Future Outlooks

In this work, we present a mathematical modelling approach to investigate the influence of environmental conditions on HGT of resistance plasmids, through the introduction of a non-local mathematical model taking into account the recipient-sensing regulating gene expression. This mechanism allowed to consider the influence of biotic and abiotic factors on plasmid spread via conjugation while still considering it as a mass action process.

The results of the numerical simulations indicate the correlation between selective pressure and plasmid persistence within a biofilm, with an increasingly more important influence of vertical gene transfer at increasing metal concentrations—up to conjugation inhibition. However, when conjugation is inhibited and the plasmid does not confer metal resistance, plasmid carriage is a metabolic burden and plasmids are bound to disappear from the bacterial population. The fitness cost of conjugation-able bacteria has very little influence, as this fraction does not quantitatively participate in the biofilm growth. This comes from the intention of this work to discriminate between plasmid carriers that express the *tra* genes or not, in order to simulate the energy optimisation at the population scale. The inclusion of $f_C$ as a separate volume fraction corresponding to a temporary state also allows to consider the non-locality of receptor sensing and its consequences on lower conjugation rates when less receptors are in the vicinity of the donor cell. The impact of conjugation on the plasmid dissemination is heightened by its interdependence with vertical gene transfer, as transconjugants are able to then divide and subsequently disseminate the plasmid in their daughter cells. This model includes the two main protection mechanisms against abiotic stressors: the diffusion barrier and genetic resistance, explaining the ecological success of the biofilm lifestyle for bacterial cells as it enhances their ability to resist external stressors.

Our model formulation correlate with the existence of the MSW (Trubenová et al. 2022) and how for a given value of toxic stressor exposition, plasmid carriage will provide or not a metabolic advantage. The addition of conjugation promotion extends this window, as it provides another way for plasmid persistence. Even in the absence of antibiotics, plasmid-borne ARG can be promoted by the means of cross-resistance or co-resistance with metals.

We conclude with a brief outline of potential model extensions for research perspectives. The model could be extended to integrate more bacterial species—as suggested in Leclerc et al. (2019)—without any modification to its structure, by adding solid-phase fractions corresponding to different species in three states: plasmid-free, plasmid carrying and conjugation-able. This could provide new information on plasmid spread while being closer to the reality by integrating incompatibility of plasmid with some species, or preferential transfer of plasmid to their own species as outlined in Dimitriu et al. (2019). Additionally, Valle et al. (2020) reported that plasmid stability increases with bacterial diversity. The inclusion of multiple species would not only increase the accuracy of the results by integrating specific antibiotics or metal toxicity towards different bacterial species, but it would also provide further insights into how the diversity of the bacterial community affects the spread of antibiotic resistance genes. Furthermore, interactions between metals and antibiotics could be added, whether it is their synergistic effect on toxicity towards cells or their deactivation by complexation (Poole 2017). Notably, this would require the introduction of a substantial amount of parameters, which can prove difficult to generate through experimental work or would have to be assumed.

Similarly to metals, antibiotics have been proven to be able to promote or inhibit plasmid transmission through conjugation (Penesyan et al. 2019; Liu et al. 2022); we neglected the effect of antibiotics on gene expression here as we focused on the metals selective pressure, but it would be possible to add the same type of functions for antibiotics effect on conjugation or potential combined effect of metals and antibiotics. This effect could be differentiated between the effect of antibiotics on *tra* genes expression and on subsequent conjugation.

From an evolutionary perspective, plasmid-borne resistance could also be compared to chromosomal resistance: while chromosomal resistance will outcompete the plasmid-encoded variant, its unability to transfer between ecotypes can cause it to disappear from a population under unfavorable environmental conditions (Carroll and Wong 2018). The presence of chromosomal resistance in a population can also affect negatively the colonisation of this population by its plasmid-encoded variant if it is costly to maintain. Host-plasmid co-evolution could also be integrated, with a mitigation of the plasmid fitness cost over generations of plasmid carriers or the migration of the beneficial plasmid-encoded genes into the chromosome.

Moreover, this model exhibits a high level of complexity due to a significant number of processes and variables involved. The outcomes of the model indicate that $\lambda _2$ has minimal impact on the final results. Conducting additional local and global sensitivity analyses would enable further streamlining of the model. Similarly, the variables $f_T$ and $f_D$, which track the evolution of the fractions of transconjugants and exhausted donors, only represent a transient state and could be neglected if the dynamics of conjugation are not of interest.

Finally, the extension of this model in 2D and 3D would certainly bring interesting perspectives by allowing to describe the emergence of resistant sub-colonies within a biofilm through conjugation bursts under beneficial conditions.

## Data Availability

The data that has been used is confidential.

## Appendix A: Local Existence and Uniqueness

Similarly to the hyperbolic integro-differential system, integral equations can also be derived for (34) and (38) by using the method of characteristics. We report here the final Volterra integral system:

A1 $$\begin{aligned} c(z_0,t)=&z_0+\int _{0}^{t} d\tau \int _{0}^{z_0} G(\textbf{f}(c(\zeta _0,\tau ),\tau ),\textbf{S}(c(\zeta _0,\tau ),\tau ))\frac{\partial c}{\partial \zeta _0}(\zeta _0,\tau )d\zeta _0, \end{aligned}$$

A2 $$\begin{aligned} \frac{\partial c}{\partial z_0}(z_0,t)=&1+\int _{0}^{t} G(\textbf{f}(c(z_0,\tau ),\tau ),\textbf{S}(c(z_0,\tau ),\tau ))\frac{\partial c}{\partial z_0}(z_0,\tau )d\tau , \end{aligned}$$

A3 $$\begin{aligned} f_i(c(z_0,t),t)=&f_{i,0}(z_0) +\int _{0}^t F_i(\textbf{f}(c(z_0,\tau ),\tau ),\textbf{S}(c(z_0,\tau ),\tau ))d\tau \nonumber \\&+ \int _{0}^t d\tau \int _{0}^{L_0}\delta _i {\bar{\nu }}_{ON}(\textbf{S}(c(z_0,\tau ),\tau )) K(c(z_0,\tau )-\zeta _0)f_F(\zeta _0,\tau )\nonumber \\&\times \frac{\partial c}{\partial \zeta _0} d\zeta _0, i\in J_f, \end{aligned}$$

A4 $$\begin{aligned} S_j(c(z_0,t),t)=&S_{j}^*(t) + D_{S,j}^{-1}\int _{z_0}^{L_0}\frac{\partial c}{\partial \eta _0}(\eta _0,t)d\eta _0 \nonumber \\&\int _{0}^{\eta _0} r_{S,j}(\textbf{f}(c(\zeta _0,t),t),\textbf{S}(c(\zeta _0,t),t))\frac{\partial c}{\partial \zeta _0}(\zeta _0,t)d\zeta _0, \nonumber \\&j\in J_S, \end{aligned}$$

where *G*, $F_i$ are the integrand functions already defined in Sect. 3.

Let us introduce the following notation

A5 $$\begin{aligned} \textbf{x}(z_0,t)= &  \textbf{f}(c(z_0,t),t),\ \textbf{x}(x_1,\ldots ,x_n), \ t > 0, \end{aligned}$$

A6 $$\begin{aligned} \textbf{s}(z_0,t)= &  \textbf{S}(c(z_0,t),t),\ \textbf{s}(s_1,\ldots ,s_m), \ t > 0, \end{aligned}$$

and consider the following positions:

A7 $$\begin{aligned}&I_i\bigg (c(z_0,\tau ),\textbf{s}(z_0,\tau ),\textbf{x}(\zeta _0,\tau ), \frac{\partial c}{\partial \zeta _0}(\zeta _0,\tau )\bigg ) \nonumber \\&\quad =\delta _i {\bar{\nu }}_{ON}(\textbf{s}(z_0,\tau )) K(c(z_0,\tau )-\zeta _0)f_F(\zeta _0,\tau ) \frac{\partial c}{\partial \zeta _0}(\zeta _0,\tau ), \end{aligned}$$

A8 $$\begin{aligned}&F_{c,1} \bigg ( \textbf{x}(\zeta _0,\tau ),\textbf{s}(\zeta _0,\tau ),\frac{\partial c}{\partial \zeta _0}(\zeta _0,\tau ) \bigg ) =G(\textbf{x}(\zeta _0,\tau ),\textbf{s}(\zeta _0,\tau ))\frac{\partial c}{\partial \zeta _0}(\zeta _0,\tau ), \end{aligned}$$

A9 $$\begin{aligned}&F_{c,2} \bigg ( \textbf{x}(z_0,\tau ),\textbf{s}(z_0,\tau ), \frac{\partial c}{\partial z_0}(z_0,\tau ) \bigg ) =G(\textbf{x}(z_0,\tau ),\textbf{s}(z_0,\tau ))\frac{\partial c}{\partial z_0}(z_0,\tau ), \end{aligned}$$

A10 $$\begin{aligned}&F_{s,j} \bigg ( \textbf{x}(\zeta _0,t),\textbf{s}(\zeta _0,t), \frac{\partial c}{\partial \eta _0}(\eta _0,t)\frac{\partial c}{\partial \zeta _0}(\zeta _0,t) \bigg ) \nonumber \\&\quad =D_{S,j}^{-1} r_{S,j}(\textbf{x}(\zeta _0,t),\textbf{s}(\zeta _0, t))\frac{\partial c}{\partial \eta _0}(\eta _0,t)\frac{\partial c}{\partial \zeta _0}(\zeta _0,t). \end{aligned}$$

Then, the integral system (A1)–(A4) is converted into the following more compact equations:

A11 $$\begin{aligned} c(z_0,t)&= z_0 + \int _{0}^{t} d\tau \int _{0}^{z_0} F_{c,1} \bigg ( \textbf{x}(\zeta _0,\tau ),\textbf{s}(\zeta _0,\tau ),\frac{\partial c}{\partial \zeta _0}(\zeta _0,\tau ) \bigg ) d\zeta _0,\nonumber \\&\quad 0 \le z_0 \le L_0, \ t>0, \end{aligned}$$

A12 $$\begin{aligned} \frac{\partial c}{\partial z_0}(z_0,t)&=1+\int _{0}^{t} F_{c,2} \bigg ( \textbf{x}(z_0,\tau ),\textbf{s}(z_0,\tau ),\frac{\partial c}{\partial z_0}(z_0,\tau ) \bigg )d\tau , \nonumber \\&\quad 0 \le z_0 \le L_0, \ t>0, \end{aligned}$$

A13 $$\begin{aligned} x_i(z_0,t)&=f_{i,0}(z_0) +\int _{0}^t F_i(\textbf{x}(z_0,\tau ),\textbf{s}(z_0,\tau ))d\tau \nonumber \\&+ \int _{0}^t d\tau \int _{0}^{L_0} I_i\bigg ({c(z_0,\tau ),\textbf{s}(z_0,\tau ),\textbf{x}}(\zeta _0,\tau ),\frac{\partial c}{\partial \zeta _0}(\zeta _0,\tau )\bigg ) d\zeta _0, \nonumber \\&\quad i\in J_f, \ 0 \le z_0 \le L_0, \ t>0,\end{aligned}$$

A14 $$\begin{aligned} s_j(z_0,t)=&S_{j}^*(t) +\int _{z_0}^{L_0} d\eta _0 \int _{0}^{\eta _0} F_{s,j} \bigg ( \textbf{x}(\zeta _0,t),\textbf{s}(\zeta _0,t),\frac{\partial c}{\partial \eta _0}(\eta _0,t),\frac{\partial c}{\partial \zeta _0}(\zeta _0,t) \bigg ) d\zeta _0, \nonumber \\&\quad j\in J_S, \ 0< z_0 < L_0, \ t>0. \end{aligned}$$

An existence and uniqueness theorem for the integral problem (A11)–(A14) can be proved in the space of the continuous functions.

### Theorem 1

Suppose that:

(i) $$c(z_0 ,t),\frac{\partial c}{\partial z_0} (z_0 ,t), x_{i} (z_0 ,t), s_{j} (z_0 ,t), \in C^0([0,\ L_0]\times [0,\ T_1])$$, $$L_0>0$$, $$T_1>0$$, $$i\in J_f$$, $$j\in J_S$$;
(ii) $$f_{i,0}(z_0) \in C^0([0,\ L_0])$$, $$L_0>0$$, $$i\in J_f$$;
(iii) $S_{j}^*(t)$, $j\in J_S$ are positive continuous functions;
(iv) $\mid c-z_0\mid \le h_{c,1}$; $\mid \frac{\partial c}{\partial z_0} -1\mid \le h_{c,2}$; $\mid x_{i} -f_{i,0}\mid \le h_{f,i}, \ i\in J_f$; $\mid s_{j}-S_{j}^*(t)\mid \le h_{s,j}, \ j\in J_S$; where $h_{c,1}$, $h_{c,2}$, $h_{f,i}$, $h_{s,j}$ are positive constants;
(v) the function $G(\textbf{x}(z_0,t),\textbf{s}(z_0,t))= \sum _{i} r_{M,i}(\textbf{x}(z_0,t),\textbf{s}(z_0,t))$ is essentially positive;
(vi) the initial biofilm thickness $L_0$ is relatively small;
(vii) $F_{c,1}$, $F_{c,2}$, $F_i, \ I_i, \ i\in J_f$, $F_{s,j}, \ j\in J_S$, are bounded and Lipschitz continuous functions with respect to their arguments
  $$\begin{aligned} &  M_{c,1}=\max | F_{c,1}|, \ M_{c,2}=\max | F_{c,2}|,\\ &  M_{F,i}=\max (| F_i| ), \ M_{I,i}=\max (| I_i| ), \ M_{i}=\max (M_{F,i},L_0 M_{I,i}), \ i\in J_f,\\ &  M_{s,j}= \max | F_{s,j}|,\ j\in J_S,\\ &  \left| F_{c,1}\left( \textbf{x},\textbf{s},\frac{\partial c}{\partial z_0} \right) -F_{c,1}\left( \tilde{\textbf{x}},\tilde{\textbf{s}},\frac{\partial {\tilde{c}}}{\partial z_0}\right) \right| \\ &  \quad \le \beta _{c,1} \Bigg [ \sum _{k=1}^{n} | x_{k} -{{\tilde{x}}}_{k} | +\sum _{k=1}^{m}| s_{k} -{{\tilde{s}}}_{k} | +| \frac{\partial c}{\partial z_0} -\frac{\partial {\tilde{c}}}{\partial z_0} | \Bigg ],\\ &  \left| F_{c,2}\left( \textbf{x},\textbf{s},\frac{\partial c}{\partial z_0} \right) -F_{c,2}\left( \tilde{\textbf{x}},\tilde{\textbf{s}},\frac{\partial {\tilde{c}}}{\partial z_0}\right) \right| \\ &  \quad \le \beta _{c,2} \Bigg [ \sum _{k=1}^{n} | x_{k} -{{\tilde{x}}}_{k} | +\sum _{k=1}^{m}| s_{k} -{{\tilde{s}}}_{k} | +| \frac{\partial c}{\partial z_0} -\frac{\partial {\tilde{c}}}{\partial z_0} | \Bigg ],\\ &  | F_i(\textbf{x},\textbf{s})-F_i(\tilde{\textbf{x}},\tilde{\textbf{s}})| \le \beta _i\left[ \sum _{k=1}^{n}| x_{k} -{{\tilde{x}}}_{k} | +\sum _{k=1}^{m}| s_{k} -{{\tilde{s}}}_{k} | \right] , \ i\in J_f,\\ &  \left| I_i\left( c, \textbf{x},\textbf{s},\frac{\partial c}{\partial z_0}\right) -I_i\left( {\tilde{c}},\tilde{\textbf{x}},\tilde{\textbf{s}}, \frac{\partial {\tilde{c}}}{\partial z_0}\right) \right| \\ &  \quad \le \beta _{I,i}\left[ | c-{{\tilde{c}}}| + \sum _{k=1}^{n}| x_{k} -{{\tilde{x}}}_{k} | +\sum _{k=1}^{m}| s_{k} -{{\tilde{s}}}_{k} | +| \frac{\partial c}{\partial z_0} -\frac{\partial {\tilde{c}}}{\partial z_0}| \right] , \ i\in J_f,\\ &  \left| F_{s,j}\left( \textbf{x},\textbf{s},\frac{\partial c}{\partial z_0} \right) - F_{s,j}\left( \tilde{\textbf{x}},\tilde{\textbf{s}},\frac{\partial {\tilde{c}}}{\partial z_0}\right) \right| \\ &  \quad \le \beta _{s,j}\Bigg [\sum _{k=1}^{n}| x_{k} -{{\tilde{x}}}_{k} | +\sum _{k=1}^{m}| s_{k} -{{\tilde{s}}}_{k} | +| \frac{\partial c}{\partial z_0} -\frac{\partial {\tilde{c}}}{\partial z_0} | \Bigg ], \ j\in J_S,\\ \end{aligned}$$
  *when* $$(z_0 ,t)\in [0,\ L_0]\times [0,\ T_1]$$ and the functions *c*, $$\frac{\partial c}{\partial z_0} $$, $$x_{i} $$, $$s_{j} $$, satisfy the assumptions (i)–(vi).

Then, integral system (A11)–(A14) has a unique solution *c*, $$\frac{\partial c}{\partial z_0} $$, $$x_{i} $$, $$s_{j} $$, $$\in C^0([0,L_0]\times [0,\ T])$$, where

$$\begin{aligned} T= &   \min \left\{ T_1,\frac{h_{f,i}}{2 M_i},\frac{h_{c,1}}{L_0 M_{c,1}},\frac{h_{c,2}}{M_{c,2}} \right\} ,\ i\in J_f. \end{aligned}$$

### Proof

Denote by $\Omega$ the space of continuous functions $c(z_0 ,t)$, $\frac{\partial c}{\partial z_0} (z_0 ,t)$, $x_{i}(z_0 ,t)$, $s_{j}(z_0 ,t)$, $z_0 \in [0, L_0]$, $t\in [0,\ T]$ and let $h_{s,j}=M_{s,j}L_0^2$. Introduce﻿ the norm

$$\begin{aligned} \begin{aligned} \left\| (c,\frac{\partial c}{\partial z_0} ,\textbf{x},\textbf{s})\right\|&=\max _{\Omega } exp(-\alpha t)\mid c\mid +\max _{\Omega } exp(-\alpha t)\mid \frac{\partial c}{\partial z_0} \mid \\&\quad +\sum _{i\in J_f}\max _{\Omega }exp(-\alpha t)\mid x_i \mid \\&\quad +\sum _{j\in J_S}\max _{\Omega }exp(-\alpha t)\mid s_j \mid , \end{aligned} \end{aligned}$$

where $\alpha$ is a positive constant. Consider the map $(c^{*},\frac{\partial c}{\partial z_0}^{*},\textbf{x}^*,\textbf{s}^*)=A(c,\frac{\partial c}{\partial z_0} ,\textbf{x},\textbf{s})$, where $(c^{*},\frac{\partial c}{\partial z_0}^{*},\textbf{x}^*,\textbf{s}^*)$= RHS of Eqs. (A11)–(A14). Let us prove that *A* maps $\Omega$ into itself. Indeed,

$$\begin{aligned} \mid c^{*}-z_0\mid\le &   M_{c,1}TL_0 \le h_{c,1},\\ \left| \frac{\partial c}{\partial z_0}^{*}-1\right|\le &   M_{c,2}T\le h_{c,2},\\ \mid x_{i}^*-f_{i,0}\mid\le &   2 M_iT \le h_{f,i},\ i\in J_f,\\ \mid s_{j}^*- S_{j}^*(t)\mid\le &   M_{s,j}L_0^2\le h_{s,j}, \ j\in J_S. \end{aligned}$$

Consider $$({{\tilde{c}}},\frac{\partial {\tilde{c}}}{\partial z_0} ,{{\tilde{{{\textbf {x}}}}}},{{\tilde{{{\textbf {s}}}}}})\in \Omega \ $$ and let $({{\tilde{c}}}^*,\frac{\partial {\tilde{c}}}{\partial z_0}^*,{{\tilde{{{\textbf {x}}}}}^*},{{\tilde{{{\textbf {s}}}}}^*}) =A({{\tilde{c}}},\frac{\partial {\tilde{c}}}{\partial z_0} ,\tilde{\mathbf {{{x}}}},{{\tilde{{{\textbf {s}}}}}})$. It is possible to obtain

$$\begin{aligned} | c^{*}-{{\tilde{c}}}^{*}| exp(-\alpha t)\le &   \frac{\beta _{c,1} L_0}{\alpha }\left\| \left( c,\frac{\partial c}{\partial z_0} ,{\textbf {x}},{\textbf {s}}\right) - \left( {{\tilde{c}}},\frac{\partial {\tilde{c}}}{\partial z_0} ,{{\tilde{{{\textbf {x}}}}}},{{\tilde{{{\textbf {s}}}}}}\right) \right\| ,\\ \left| \frac{\partial c}{\partial z_0}^{*}-\frac{\partial {\tilde{c}}}{\partial z_0}^{*}\right| exp(-\alpha t)\le &   \frac{\beta _{c,2}}{\alpha }\left\| \left( c,\frac{\partial c}{\partial z_0} ,{\textbf {x}},{\textbf {s}}\right) - \left( {{\tilde{c}}},\frac{\partial {\tilde{c}}}{\partial z_0} ,{{\tilde{{{\textbf {x}}}}}},{{\tilde{{{\textbf {s}}}}}}\right) \right\| ,\\ | x_{i}^{*}-{{\tilde{x}}}_{i}^{*}| exp(-\alpha t)\le &   \left( \frac{\beta _i}{\alpha }+\frac{\beta _{I,i}L_0}{\alpha }\right) \left\| \left( c,\frac{\partial c}{\partial z_0} ,{\textbf {x}},{\textbf {s}}\right) - \left( {{\tilde{c}}},\frac{\partial {\tilde{c}}}{\partial z_0} ,{{\tilde{{{\textbf {x}}}}}},{{\tilde{{{\textbf {s}}}}}}\right) \right\| , \ i\in J_f,\\ | s_{j}^{*}-{{\tilde{s}}}_{j}^{*}| exp(-\alpha t)\le &   \beta _{s,j} L_0^2\left\| \left( c,\frac{\partial c}{\partial z_0} ,{\textbf {x}},{\textbf {s}}\right) - \left( {{\tilde{c}}},\frac{\partial {\tilde{c}}}{\partial z_0} ,{{\tilde{{{\textbf {x}}}}}},{{\tilde{{{\textbf {s}}}}}}\right) \right\| , \ j\in J_S, \end{aligned}$$

Hence,

$$\begin{aligned} \begin{aligned}&\left\| \left( c^{*},\frac{\partial c}{\partial z_0}^{*},{\textbf {x}}^*,{\textbf {s}}^*\right) -\left( {{\tilde{c}}}^*,\frac{\partial {\tilde{c}}}{\partial z_0}^*,{{\tilde{{{\textbf {x}}}}}^*},{{\tilde{{{\textbf {s}}}}}^*}\right) \right\| \le \Lambda \left\| \left( c,\frac{\partial c}{\partial z_0} ,{\textbf {x}},{\textbf {s}}\right) \right. \\&\left. \quad - \left( {{\tilde{c}}},\frac{\partial {\tilde{c}}}{\partial z_0} ,{{\tilde{{{\textbf {x}}}}}},{{\tilde{{{\textbf {s}}}}}}\right) \right\| , \end{aligned} \end{aligned}$$

where

$$\begin{aligned} \Lambda= &   \Lambda _1+\Lambda _2,\\ \Lambda _1= &   \frac{\beta _{c,1} L_0}{\alpha } + \frac{\beta _{c,2}}{\alpha } +\sum _{i\in J_f} \left( \frac{\beta _i}{\alpha }+\frac{\beta _{I,i}L_0}{\alpha }\right) \\ \Lambda _2= &   L_0^2 \sum _{j\in J_S} \beta _{S,j}. \end{aligned}$$

The positive constant $\alpha$ was not specified earlier. Therefore, it can be chosen very large so that $\Lambda _1$ becomes very small, say $<\epsilon , \forall \epsilon >0$, and $L_0$ small enough so that

$$\begin{aligned} L_0^2 \le (1-\epsilon ) (\sum _{j\in J_S} \beta _{S,j})^{-1} \end{aligned}$$

Then $\Lambda <1$, and Theorem 1 is proven. $\square$

## References

[CR1] Abe K, Nomura N, Suzuki S (2020) Biofilms: hot spots of horizontal gene transfer (HGT) in aquatic environments, with a focus on a new HGT mechanism. FEMS Microbiol Ecol. 10.1093/femsec/fiaa031 10.1093/femsec/fiaa031 PMC7189800 32109282

[CR2] Alonso-del Valle A, León-Sampedro R, Rodríguez-Beltrán J, DelaFuente J, Hernández-García M, Ruiz-Garbajosa P, Cantón R, Peña-Miller R, Millán AS (2020) The distribution of plasmid fitness effects explains plasmid persistence in bacterial communities. bioRxiv. 10.1101/2020.08.01.230672 10.1038/s41467-021-22849-y PMC8113577 33976161

[CR3] Arya S, Williams A, Reina SV, Knapp CW, Kreft J-U, Hobman JL, Stekel DJ (2021) Towards a general model for predicting minimal metal concentrations co-selecting for antibiotic resistance plasmids. Environ Pollut 275:116602. 10.1016/j.envpol.2021.116602 33582634 10.1016/j.envpol.2021.116602

[CR4] Baker-Austin C, Wright MS, Stepanauskas R, McArthur JV (2006) Co-selection of antibiotic and metal resistance. Trends Microbiol 14(4):176–182. 10.1016/j.tim.2006.02.006 16537105 10.1016/j.tim.2006.02.006

[CR5] Baltrus DA (2013) Exploring the costs of horizontal gene transfer. Trends Ecol Evolut 28(8):489–495. 10.1016/j.tree.2013.04.002 10.1016/j.tree.2013.04.002 23706556

[CR6] Barkay T, Miller SM, Summers AO (2003) Bacterial mercury resistance from atoms to ecosystems. FEMS Microbiol Rev 27(2–3):355–384. 10.1016/S0168-6445(03)00046-9 12829275 10.1016/S0168-6445(03)00046-9

[CR7] Beaber JW, Hochhut B, Waldor MK (2004) SOS response promotes horizontal dissemination of antibiotic resistance genes. Nature 427(6969):72–74. 10.1038/nature02241 14688795 10.1038/nature02241

[CR8] Bose B, Grossman AD (2011) Regulation of horizontal gene transfer in *Bacillus subtilis* by activation of a conserved site-specific protease. J Bacteriol 193(1):22–29. 10.1128/JB.01143-10 21036995 10.1128/JB.01143-10 PMC3019953

[CR9] Cai P, Chen Q, Du W, Yang S, Li J, Cai H, Zhao X, Sun W, Xu N, Wang J (2023) Deciphering the dynamics of metal and antibiotic resistome profiles under different metal (loid) contamination levels. J Hazard Mater 455:131567 37167868 10.1016/j.jhazmat.2023.131567

[CR10] Campos M, Capilla R, Naya F, Futami R, Coque T, Moya A, Fernandez-Lanza V, Cantón R, Sempere JM, Llorens C, Baquero F (2019) Simulating multilevel dynamics of antimicrobial resistance in a membrane computing model. mBio 10(1):02460–18. 10.1128/mBio.02460-18 10.1128/mBio.02460-18 PMC6355984 30696743

[CR11] Carroll AC, Wong A (2018) Plasmid persistence: costs, benefits, and the plasmid paradox. Can J Microbiol 64(5):293–304. 10.1139/cjm-2017-0609 29562144 10.1139/cjm-2017-0609

[CR12] Cazer CL, Ducrot L, Volkova VV, Gröhn YT (2017) Monte Carlo simulations suggest current chlortetracycline drug-residue based withdrawal periods would not control antimicrobial resistance dissemination from feedlot to slaughterhouse. Front Microbiol 8:1753 29033901 10.3389/fmicb.2017.01753 PMC5627025

[CR13] Chambless JD, Hunt SM, Stewart PS (2006) A three-dimensional computer model of four hypothetical mechanisms protecting biofilms from antimicrobials. Appl Environ Microbiol 72(3):2005–2013. 10.1128/AEM.72.3.2005-2013.2006 16517649 10.1128/AEM.72.3.2005-2013.2006 PMC1393201

[CR14] Chen J, Li J, Zhang H, Shi W, Liu Y (2019) Bacterial heavy-metal and antibiotic resistance genes in a copper tailing dam area in Northern China. Front Microbiol 10:1916 31481945 10.3389/fmicb.2019.01916 PMC6710345

[CR15] Ciofu O, Moser C, Jensen PO, Høiby N (2022) Tolerance and resistance of microbial biofilms. Nat Rev Microbiol. 10.1038/s41579-022-00682-4 10.1038/s41579-022-00682-4 35115704

[CR16] D’Acunto B, Frunzo L (2011) Qualitative analysis and simulations of a free boundary problem for multispecies biofilm models. Math Comput Model 53(9–10):1596–1606

[CR17] D’Acunto B, Frunzo L, Luongo V, Mattei MR (2019) Modeling heavy metal sorption and interaction in a multispecies biofilm. Mathematics 7(9):781. 10.3390/math7090781

[CR18] D’Acunto B, Frunzo L, Luongo V, Mattei MR, Tenore A (2021) Free boundary problem for the role of planktonic cells in biofilm formation and development. Z Angew Math Phys 72(4):149. 10.1007/s00033-021-01561-3

[CR19] Dimitriu T, Marchant L, Buckling A, Raymond B (2019) Bacteria from natural populations transfer plasmids mostly towards their kin. Proc R Soc B Biol Sci 286(1905):20191110. 10.1098/rspb.2019.1110 10.1098/rspb.2019.1110 PMC6599995 31238848

[CR20] Eberl H, Association IW (eds) (2006) Mathematical modeling of biofilms, 1 edn. Scientific and technical report/IWA, IWA Publ, London, vol 18

[CR21] Enne VI, Bennett PM, Livermore DM, Hall LMC (2004) Enhancement of host fitness by the sul2-coding plasmid p9123 in the absence of selective pressure. J Antimicrob Chemother 53(6):958–963. 10.1093/jac/dkh217 15102746 10.1093/jac/dkh217

[CR22] Evans G (1993) Practical numerical integration. Wiley, Chichester

[CR23] Flemming H-C, Wingender J (2010) The biofilm matrix. Nat Rev Microbiol 8(9):623–633. 10.1038/nrmicro2415 20676145 10.1038/nrmicro2415

[CR24] Flemming H-C, Wingender J, Szewzyk U, Steinberg P, Rice SA, Kjelleberg S (2016) Biofilms: an emergent form of bacterial life. Nat Rev Microbiol 14(9):563–575. 10.1038/nrmicro.2016.94 27510863 10.1038/nrmicro.2016.94

[CR25] Freese PD, Korolev KS, Jiménez JI, Chen IA (2014) Genetic drift suppresses bacterial conjugation in spatially structured populations. Biophys J 106(4):944–954. 10.1016/j.bpj.2014.01.012 24559997 10.1016/j.bpj.2014.01.012 PMC3944618

[CR26] Friedman A, Kao C-Y (2014) Mathematical modeling of biological processes. Springer, Cham

[CR27] Frost LS, Koraimann G (2010) Regulation of bacterial conjugation: balancing opportunity with adversity. Future Microbiol 5(7):1057–1071. 10.2217/fmb.10.70 20632805 10.2217/fmb.10.70

[CR28] Frunzo L, Mattei MR (2017) Qualitative analysis of the invasion free boundary problem in biofilms. Ricerche Mat 66:171–188

[CR29] Gaebler HJ, Eberl HJ (2018) A simple model of biofilm growth in a porous medium that accounts for detachment and attachment of suspended biomass and their contribution to substrate degradation. Eur J Appl Math 29(6):1110–1140

[CR30] Ghasemi M, Hense BA, Eberl HJ, Kuttler C (2018) Simulation-based exploration of quorum sensing triggered resistance of biofilms to antibiotics. Bull Math Biol 80(7):1736–1775. 10.1007/s11538-018-0433-3 29691717 10.1007/s11538-018-0433-3

[CR31] Gothwal R, Thatikonda S (2018) Mathematical model for the transport of fluoroquinolone and its resistant bacteria in aquatic environment. Environ Sci Pollut Res 25(21):20439–20452. 10.1007/s11356-017-9848-x 10.1007/s11356-017-9848-x 28780691

[CR32] Guo J, Li J, Chen H, Bond PL, Yuan Z (2017) Metagenomic analysis reveals wastewater treatment plants as hotspots of antibiotic resistance genes and mobile genetic elements. Water Res 123:468–478. 10.1016/j.watres.2017.07.002 28689130 10.1016/j.watres.2017.07.002

[CR33] Györi I (1988) The method of lines for the solutions of some nonlinear partial differential equations. Comput Math Appl 15(6–8):635–658

[CR34] Hill WR, Larsen IL (2005) Growth dilution of metals in microalgal biofilms. Environ Sci Technol 39(6):1513–1518. 10.1021/es049587y 15819203 10.1021/es049587y

[CR35] Kneis D, Hiltunen T, Heß S (2019) A high-throughput approach to the culture-based estimation of plasmid transfer rates. Plasmid 101:28–34. 10.1016/j.plasmid.2018.12.003 30599142 10.1016/j.plasmid.2018.12.003

[CR36] Knopoff DA, Sánchez Sansó JM (2016) A kinetic model for horizontal transfer and bacterial antibiotic resistance. Int J Biomath 10(04):1750051. 10.1142/S1793524517500516

[CR37] Koraimann G, Wagner MA (2014) Social behavior and decision making in bacterial conjugation. Front Cell Infect Microbiol 4:54 24809026 10.3389/fcimb.2014.00054 PMC4010749

[CR38] Lambertsen LM, Molin S, Kroer N, Thomas CM (2004) Transcriptional regulation of pWW0 transfer genes in *Pseudomonas putida* KT2440. Plasmid 52(3):169–181. 10.1016/j.plasmid.2004.06.005 15518874 10.1016/j.plasmid.2004.06.005

[CR39] Leclerc QJ, Lindsay JA, Knight GM (2019) Mathematical modelling to study the horizontal transfer of antimicrobial resistance genes in bacteria: current state of the field and recommendations. J R Soc Interface 16(157):20190260. 10.1098/rsif.2019.0260 31409239 10.1098/rsif.2019.0260 PMC6731517

[CR40] Li B, Qiu Y, Song Y, Lin H, Yin H (2019) Dissecting horizontal and vertical gene transfer of antibiotic resistance plasmid in bacterial community using microfluidics. Environ Int 131:105007. 10.1016/j.envint.2019.105007 31326825 10.1016/j.envint.2019.105007

[CR41] Lide DR (ed) (2005) CRC handbook of chemistry and physics: a ready-reference of chemical and physical data, 85th Ed. vol 127, pp 4542–4542. 10.1021/ja041017a

[CR42] Lin H, Jiang L, Li B, Dong Y, He Y, Qiu Y (2019) Screening and evaluation of heavy metals facilitating antibiotic resistance gene transfer in a sludge bacterial community. Sci Total Environ 695:133862. 10.1016/j.scitotenv.2019.133862 31425984 10.1016/j.scitotenv.2019.133862

[CR43] Liu G, Thomsen LE, Olsen JE (2022) Antimicrobial-induced horizontal transfer of antimicrobial resistance genes in bacteria: a mini-review. J Antimicrob Chemother 77(3):556–567. 10.1093/jac/dkab450 34894259 10.1093/jac/dkab450

[CR44] Mattei MR, Frunzo L, D’Acunto B, Pechaud Y, Pirozzi F, Esposito G (2018) Continuum and discrete approach in modeling biofilm development and structure: a review. J Math Biol 76(4):945–1003. 10.1007/s00285-017-1165-y 28741178 10.1007/s00285-017-1165-y

[CR45] Mohanty RK (2012) A combined arithmetic average discretization and TAGE iterative method for non-linear two point boundary value problems with a source function in integral form. Differ Equ Dyn Syst 20(4):423–440. 10.1007/s12591-012-0140-8

[CR46] Nanda M, Kumar V, Sharma DK (2019) Multimetal tolerance mechanisms in bacteria: the resistance strategies acquired by bacteria that can be exploited to ‘clean-up’ heavy metal contaminants from water. Aquat Toxicol 212:1–10. 10.1016/j.aquatox.2019.04.011 31022608 10.1016/j.aquatox.2019.04.011

[CR47] Newbury A, Dawson B, Klümper U, Hesse E, Castledine M, Fontaine C, Buckling A, Sanders D (2022) Fitness effects of plasmids shape the structure of bacteria-plasmid interaction networks. Proc Natl Acad Sci 119(22):2118361119. 10.1073/pnas.2118361119 10.1073/pnas.2118361119 PMC9295774 35613058

[CR48] Pal C, Bengtsson-Palme J, Kristiansson E, Larsson DGJ (2015) Co-occurrence of resistance genes to antibiotics, biocides and metals reveals novel insights into their co-selection potential. BMC Genom 16(1):964. 10.1186/s12864-015-2153-5 10.1186/s12864-015-2153-5 PMC4650350 26576951

[CR49] Pallares-Vega R, Macedo G, Brouwer MSM, Hernandez Leal L, Maas P, Loosdrecht MCM, Weissbrodt DG, Heederik D, Mevius D, Schmitt H (2021) Temperature and nutrient limitations decrease transfer of conjugative IncP-1 plasmid pKJK5 to wild *Escherichia coli* strains. Front Microbiol 12:656250 34349732 10.3389/fmicb.2021.656250 PMC8326584

[CR50] Penesyan A, Nagy SS, Kjelleberg S, Gillings MR, Paulsen IT (2019) Rapid microevolution of biofilm cells in response to antibiotics. NPJ Biofilms Microbiomes 5(1):1–14. 10.1038/s41522-019-0108-3 31728201 10.1038/s41522-019-0108-3 PMC6834608

[CR51] Poole K (2017) At the nexus of antibiotics and metals: the impact of Cu and Zn on antibiotic activity and resistance. Trends Microbiol 25(10):820–832. 10.1016/j.tim.2017.04.010 28526548 10.1016/j.tim.2017.04.010

[CR52] Pruden A (2014) Balancing water sustainability and public health goals in the face of growing concerns about antibiotic resistance. Environ Sci Technol 48(1):5–14. 10.1021/es403883p 24279909 10.1021/es403883p

[CR53] Rahman KA, Sudarsan R, Eberl HJ (2015) A mixed-culture biofilm model with cross-diffusion. Bull Math Biol 77(11):2086–2124. 10.1007/s11538-015-0117-1 26582360 10.1007/s11538-015-0117-1

[CR54] Roberts MC (2005) Update on acquired tetracycline resistance genes. FEMS Microbiol Lett 245(2):195–203. 10.1016/j.femsle.2005.02.034 15837373 10.1016/j.femsle.2005.02.034

[CR55] Russo F, Tenore A, Mattei MR, Frunzo L (2022) Multiscale modelling of the start-up process of anammox-based granular reactors. Math Biosci Eng 19(10):10374–10406 36031999 10.3934/mbe.2022486

[CR56] Russo F, Tenore A, Mattei MR, Frunzo L (2023) A mathematical study of metal biosorption on algal-bacterial granular biofilms. Bull Math Biol 85(7):63 37269488 10.1007/s11538-023-01168-x PMC10239425

[CR57] Savage VJ, Chopra I, O’Neill AJ (2013) *Staphylococcus aureus* biofilms promote horizontal transfer of antibiotic resistance. Antimicrob Agents Chemother 57(4):1968–1970. 10.1128/AAC.02008-12 23357771 10.1128/AAC.02008-12 PMC3623343

[CR58] Shi X, Xia Y, Wei W, Ni B-J (2022) Accelerated spread of antibiotic resistance genes (ARGs) induced by non-antibiotic conditions: roles and mechanisms. Water Res 224:119060. 10.1016/j.watres.2022.119060 36096030 10.1016/j.watres.2022.119060

[CR59] Singh PK, Ramachandran G, Ramos-Ruiz R, Peiró-Pastor R, Abia D, Wu LJ, Meijer WJJ (2013) Mobility of the native *Bacillus subtilis* conjugative plasmid pLS20 is regulated by intercellular signaling. PLoS Genet 9(10):1003892. 10.1371/journal.pgen.1003892 10.1371/journal.pgen.1003892 PMC3814332 24204305

[CR60] Smillie C, Garcillán-Barcia MP, Francia MV, Rocha EPC, Cruz F (2010) Mobility of plasmids. Microbiol Mol Biol Rev 74(3):434–452. 10.1128/MMBR.00020-10 20805406 10.1128/MMBR.00020-10 PMC2937521

[CR61] Stalder T, Top E (2016) Plasmid transfer in biofilms: a perspective on limitations and opportunities. NPJ Biofilms Microbiomes 2(1):1–5. 10.1038/npjbiofilms.2016.22 28480050 10.1038/npjbiofilms.2016.22 PMC5416938

[CR62] Sun F, Xu Z, Fan L (2021) Response of heavy metal and antibiotic resistance genes and related microorganisms to different heavy metals in activated sludge. J Environ Manag 300:113754 10.1016/j.jenvman.2021.113754 34543965

[CR63] Suzuki S, Kimura M, Agusa T, Rahman HM (2012) Vanadium accelerates horizontal transfer of tet (m) gene from marine photobacterium to *Escherichia coli*. FEMS Microbiol Lett 336(1):52–56 22889204 10.1111/j.1574-6968.2012.02653.x

[CR64] Szomolay B, Klapper I, Dockery J, Stewart PS (2005) Adaptive responses to antimicrobial agents in biofilms. Environ Microbiol 7(8):1186–1191. 10.1111/j.1462-2920.2005.00797.x 16011755 10.1111/j.1462-2920.2005.00797.x

[CR65] Tenore A, Russo F, Mattei MR, D’Acunto B, Collins G, Frunzo L (2021) Multiscale modelling of de novo anaerobic granulation. Bull Math Biol 83(12):122. 10.1007/s11538-021-00951-y 34741191 10.1007/s11538-021-00951-y PMC8571262

[CR66] Tenore A, Mattei MR, Frunzo L (2021) Modelling the ecology of phototrophic-heterotrophic biofilms. Commun Nonlinear Sci Numer Simul 94:105577

[CR67] Trubenová B, Roizman D, Moter A, Rolff J, Regoes RR (2022) Population genetics, biofilm recalcitrance, and antibiotic resistance evolution. Trends Microbiol. 10.1016/j.tim.2022.02.005 10.1016/j.tim.2022.02.005 35337697

[CR68] Vogwill T, MacLean RC (2015) The genetic basis of the fitness costs of antimicrobial resistance: a meta-analysis approach. Evol Appl 8(3):284–295. 10.1111/eva.12202 25861386 10.1111/eva.12202 PMC4380922

[CR69] Volkova VV, Lu Z, Lanzas C, Scott HM, Gröhn YT (2013) Modelling dynamics of plasmid-gene mediated antimicrobial resistance in enteric bacteria using stochastic differential equations. Sci Rep 3(1):2463. 10.1038/srep02463 23982723 10.1038/srep02463 PMC3755285

[CR70] Wan Z, Varshavsky J, Teegala S, McLawrence J, Goddard NL (2011) Measuring the rate of conjugal plasmid transfer in a bacterial population using quantitative PCR. Biophys J 101(1):237–244. 10.1016/j.bpj.2011.04.054 21723834 10.1016/j.bpj.2011.04.054 PMC3127179

[CR71] Wang R, Chen M, Feng F, Zhang J, Sui Q, Tong J, Wei Y, Wei D (2017) Effects of chlortetracycline and copper on tetracyclines and copper resistance genes and microbial community during swine manure anaerobic digestion. Biores Technol 238:57–69. 10.1016/j.biortech.2017.03.134 10.1016/j.biortech.2017.03.134 28432950

[CR72] Wright GD (2010) The antibiotic resistome. Expert Opin Drug Discov 5(8):779–788. 10.1517/17460441.2010.497535 22827799 10.1517/17460441.2010.497535

[CR73] Xu S, Yang J, Yin C, Zhao X (2018) The dominance of bacterial genotypes leads to susceptibility variations under sublethal antibiotic pressure. Future Microbiol 13(2):165–185. 10.2217/fmb-2017-0070 29260580 10.2217/fmb-2017-0070

[CR74] Xu Y, Tan L, Li Q, Zheng X, Liu W (2022) Sublethal concentrations of heavy metals Cu and Zn can induce the emergence of bacterial multidrug resistance. Environ Technol Innov. 10.1016/j.eti.2022.102379

[CR75] Zhang T, Li B (2011) Occurrence, transformation, and fate of antibiotics in municipal wastewater treatment plants. Crit Rev Environ Sci Technol 41(11):951–998. 10.1080/10643380903392692

[CR76] Zhang Y, Gu AZ, Cen T, Li X, He M, Li D, Chen J (2018) Sub-inhibitory concentrations of heavy metals facilitate the horizontal transfer of plasmid-mediated antibiotic resistance genes in water environment. Environ Pollut 237:74–82. 10.1016/j.envpol.2018.01.032 29477117 10.1016/j.envpol.2018.01.032

[CR77] Zhong X, Droesch J, Fox R, Top EM, Krone SM (2012) On the meaning and estimation of plasmid transfer rates for surface-associated and well-mixed bacterial populations. J Theor Biol 294:144–152. 10.1016/j.jtbi.2011.10.034 22085738 10.1016/j.jtbi.2011.10.034 PMC3346278

[CR78] Zwanzig M, Harrison E, Brockhurst MA, Hall JPJ, Berendonk TU, Berger U (2019) Mobile compensatory mutations promote plasmid survival. mSystems 4(1):00186–18. 10.1128/mSystems.00186-18 10.1128/mSystems.00186-18 PMC6446977 30944871

